# APOBEC-1 cofactors regulate APOBEC3-induced mutations in hepatitis B virus

**DOI:** 10.1128/jvi.01879-24

**Published:** 2025-01-27

**Authors:** Zhigang Chen, Thomas L. Eggerman, Alexander V. Bocharov, Irina N. Baranova, Tatyana G. Vishnyakova, Amy P. Patterson

**Affiliations:** 1 Department of Laboratory Medicine, Clinical Center, National Institutes of Health 378162 , Bethesda, Maryland, USA; 2 Division of Diabetes, Endocrinology and Metabolic Diseases, National Institute of Diabetes and Digestive and Kidney Diseases 377213 , Bethesda, Maryland, USA; 3 National Heart, Lung and Blood Institute, National Institutes of Health 35035 , Bethesda, Maryland, USA; Lerner Research Institute, Cleveland Clinic, Cleveland, Ohio, USA

**Keywords:** cofactor, hepatitis B virus, hypermutation, regulation, APOBEC3, hnRNP, mutation

## Abstract

APOBEC3 proteins (A3s) play an important role in host innate immunity against viruses and DNA mutations in cancer. A3s-induced mutations in both viral and human DNA genomes vary significantly from non-lethal mutations in viruses to localized hypermutations, such as kataegis in cancer. How A3s are regulated remains largely unknown. Since A3s exist in complexes and belong to the same family as APOBEC-1 (A1), which requires cofactors to be functional, we investigated the role of A1 cofactors and other A3 potentially associated hnRNPs on A3 mutational activity using hepatitis B virus (HBV) cellular replication as a model. We found that A1-associated cofactors and other hnRNPs were involved in A3 mutational activity regulation, and their regulatory effect was dependent on the strength of A3 association with hnRNPs with an order of A3C > 3G>3B and A1 cofactors > other hnRNPs. A1 cofactors had a strong protein interaction with A3 and significantly increased A3 mutational activity by co-expression. Endogenous gene expression knockdown by siRNA had the opposite decrease effect. Disruption of the protein interactions between A3 and hnRNPs through A3G and A3B mutagenesis decreased A3 mutational activity significantly, even to a level of near total loss, indicating that A1 cofactors and hnRNPs are required for A3 mutational activity. HBV genome-wide mutation analyses showed that A1 cofactors significantly increased A3C accessibility to HBV (−)DNA and A3C-induced mutational efficiency to generate kataegis-like hypermutation. These data demonstrate that A1 cofactors and hnRNPs are closely associated with A3s and may play an important regulatory role under physiological conditions.

**IMPORTANCE:**

As human host restriction factors, A3s play an important role against viral infections. A3s are also major mutagenic drivers of cancer. However, why A3-induced mutations vary significantly from non-lethal mutations in virus to localized hypermutations in cancer remains unknown. We found that A1 cofactor and other hnRNPs are not only associated with A3 complexes but also play important regulatory roles in A3-induced mutation activities. A1 cofactors like GRY-RBP significantly increased A3 accessibility and mutational efficiency to its single-strand DNA substrate during HBV reverse transcription to generate hypermutations. Disruption of the A3 protein association with hnRNPs by A3 mutagenesis diminished A3 mutational activity. This finding not only reveals a regulatory mechanism for A3-induced mutation but also indicates that A3-associated cellular factors can be a potential target for regulating A3-induced mutation for cancer therapeutics.

## INTRODUCTION

Apolipoprotein B mRNA-editing enzyme catalytic polypeptide-like-3 (APOBEC3) cytidine deaminases catalyze cytidine-to-uridine mutations in both viral and cellular genomes (1–3). The APOBEC3 (A3) protein family consists of seven members of A3A–H, sharing the common characteristic of cytidine deaminase activity on single-stranded DNA (ssDNA) (1). Among them, A3G was first identified as a cellular factor against human immunodeficiency virus-1 (HIV-1) based on the significant differential infectivity of Vif-defective HIV-1 virus in cultured cells with different A3G expression levels (4). Later, multiple A3s have been found to perform essential roles in innate immunity against other viruses including HIV-1, human T-cell leukemia virus type 1 (HTLV-1), hepatitis B virus (HBV), adeno-associated virus (AAV), human Papillomavirus (HPV), and others (1). Recently, A3s have also emerged as causes of somatic mutagenesis in cancer from large human genome sequencing analyses (5–7). A3 enzymes, especially A3B are associated with a distinct mutational signature in the genomes of many cancers and have been described as a major cause of mutations in approximately 15% of sequenced human tumors (5, 6). A3-associated local hypermutation, namely kataegis, has been observed in 55% of breast tumors (8). A3 mutations have been associated clinically with sub-clonal diversity in tumors (5, 9), tumor recurrence (10), metastasis (10), treatment resistance (7, 11), and improved survival (7, 8).

The inhibitory function of A3 proteins against viruses has been extensively investigated (1). A3s can hypermutate various viral genomes. However, A3 mutational efficiency varies significantly even among different mutation reactions of the same viral genome. It has been reported that A3G can hypermutate the HIV-1 genome to such an extent that they cannot produce infectious progeny virus (12). A3G can also cause sub-lethal mutations to generate new HIV-1 variants (13). Similarly, HBV viral genomes can be mutated by multiple A3s. However, the C-to-T mutational efficiency in different mutation reactions as captured by HBV genome clonal sequencing analyses varies from 0.4% to 27.9% with A3C (14). On the other hand, when human cancer cell lines were cultured for extended periods to investigate ongoing patterns of mutation generation, signatures of A3-induced genome mutations displayed substantial fluctuation in the mutation rate over time with episodic bursts of mutations (15). The initiating factors for the bursts of mutations are unknown and not associated with the expression levels of A3s. Taken together, these data indicate that A3 expression levels are not the only determining factor and that A3 enzyme mutational activity can be dramatically upregulated by the presence of other unknown cellular factor(s). How A3 enzyme activities are regulated to the point of inducing hypermutations under physiological conditions are unknown.

A3s are zinc-dependent deaminases that mutate deoxycytidine (dC) to deoxyuridine (dU) on single-stranded DNA (ssDNA) (16). Single-stranded DNA is only temporarily available when viral RNA genomes are reversely transcribed in viral particles or when DNA genomes are transcribed into RNA or replicated in cancer cells (7, 17). An A3 mutational reaction requires A3 enzyme access to the ssDNA substrate in the viral reverse-transcription or DNA replication complexes (16). Making ssDNAs available for A3-induced mutations is a complicated process, and other cellular proteins could be potentially involved because A3 mutational activity is closely associated with the viral reverse transcriptional process as reported for HBV (14). APOBEC-1 (A1) is the founder of APOBEC family that plays a critical role in apolipoprotein B metabolism (1). A1 is an RNA-editing enzyme and causes a C-to-U mutation on one specific cytidine at site 6,666 of apolipoprotein B mRNA. However, A1 alone is not functional, and it requires cofactors to form a multicomponent editing enzyme complex that enables A1 to access the site-specific cytidine and perform the C-to-U mutations under physiological conditions (18, 19). Multiple protein cofactors have been identified to associate with the A1 editing complex including ACF, CUGBP2, GRY-RBP (hnRNP-Q), KSRP, hnRNP-C1, ABBP1 (hnRNP-AB), ABBP2, and BAG4. Recently, RBM47 has also been identified as essential tissue specific cofactor for A1 function (20). These A1 cofactors are coordinately expressed with A1 and play an important regulatory role in A1 mutational activity *in vivo* (18, 21).

A1 and A3 belong to the same APOBEC family. The cofactor requirement of A1 suggests that similar cellular cofactors might be involved in A3 function under physiological conditions (22). In fact, A3s bind quickly to RNAs to form ribonucleoprotein (RNP) complexes once translated in the cellular cytoplasm, and multiple hnRNPs have been identified in the A3 complexes through A3 immuno-precipitation (16, 23–26). Intracellular A3G exists either as an enzymatically active noncomplexed component in a low molecular mass (LMM) form or as an enzymatically inactive high-molecular mass (HMM) form, depending on differential binding to RNA and multiple RNA-binding proteins in the A3 complexes (16, 23–25). It is reported that these RNAs are required for the complex formation or even A3 functional structure formation as in the case of A3H and that RNAs actively participate in the regulation of A3H mutational activity under natural conditions (27, 28). In addition, heat shock proteins (Hsp), especially Hsp90 have been found to be a A3 molecular chaperone and stimulate A3 mutation activity on HBV viral DNA (25). Taken together, A3 proteins are intracellular complexes tightly associated with multiple cellular factors, and overall, hnRNPs are the major component in the A3 complexes. However, whether these hnRNPs have any potential role in A3 enzyme mutational activity is unknown. In this study, we investigated the role of A1 cofactors and other representative hnRNPs on A3 mutational activity using HBV cellular replication as a model. We found that hnRNPs are required for A3 mutational function and over-expression of A1 cofactors like GRY-RBP can dramatically stimulate A3 mutation efficiency to generate kataegis-like hypermutation, indicating that A1 cofactors and other RNPs are involved in A3 mutational function and may play an important regulatory role under physiological conditions.

## RESULTS

### A1 cofactors have a strong protein interaction with A3s

**Fig 1:**
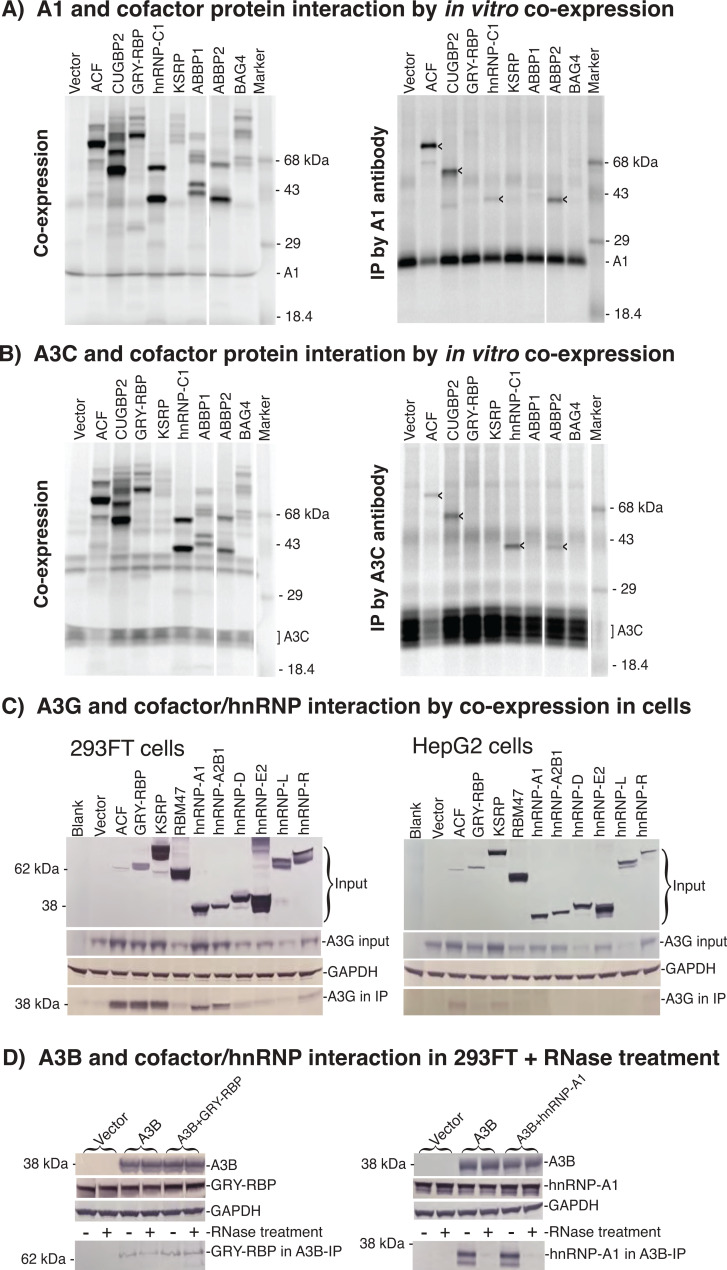
A3s have protein interactions with A1 cofactors and other hnRNPs. (**A**) APOBEC-1 (A1) or (**B**) A3C *in vitro* protein interaction analyses with A1 cofactors. A1 or A3C was co-expressed with A1 cofactors in an *in vitro*-coupled transcription/translation system in the presence of ^35^S-methionine. The A1 or A3C protein complexes formed during the *in vitro* translation were immunoprecipitated by an antibody against A1 or A3C. The precipitated protein complexes were separated by a 12% SDS-PAGE denaturing gel and the ^35^S-labeled proteins were detected by a PhosphoImager. The results are presented as co-expression protein content in the left panel and proteins detected In the IP complexes in the right panel. (**C**) A3G cellular protein interaction analyses with A1 cofactors and hnRNPs. A3G was co-transfected with representative A1 cofactors or hnRNAs in 293 FT or HepG2 cells. The resultant A3G complexes in the cell lysates were immunoprecipitated by an antibody against the Flag tag in the C-terminals of A1 cofactors or hnRNPs. The IP complexes were separated by a 10% SDS-PAGE denaturing gel, and the protein interactions were evaluated by the detection of A3G in the IPs using an antibody against A3G. (**D**) A3B direct or indirect cellular protein interaction analyses by rNase treatment. A3B was transfected with or without GRY-RBP or hnRNP-A1 in 293 FT cells. The resultant A3B complexes in the cell lysates were immunoprecipitated by an antibody against A3B with or without RNase pre-treatment. GRY-RBP and hnRNP-A1 were analyzed for their presence in the A3B complex IPs by western blotting using an antibody against GRY-RBP or hnRNP-A1.

A1 cofactors play important regulatory roles in A1-induced site-specific apoB mRNA editing. A1 over-expression disrupted its editing fidelity and led to hypermutations with a pattern-like A3s (1, 22). Human A1 over-expression-induced hypermutations were further increased by co-expression of the A1 cofactor ACF but were decreased by A1 mutagenesis that weakened A1 protein interaction with ACF (1, 29). As A1 and A3 belong to the same APOBEC family, these data suggest that A1 cofactors could also be involved in A3 mutational activity. To investigate this possibility, A3C and A1 were co-expressed with each individual A1 cofactor by *in vitro* TNT quick-coupled transcription/translation systems. The potential A1 cofactor protein interactions with A3C and A1 were evaluated in parallel by immunoprecipitation (IP) using an antibody against A3C or A1. As shown in Fig. 1A and B, A3C and A1 had almost identical protein interactions with ACF, CUGBP2, hnRNP-C1, and ABBP2, although the intensity of individual proteins in the IP pellets varied, suggesting that A1 cofactors also interact with A3C.

To evaluate the biological relevance of A1 cofactors to A3, we investigated A1 cofactor protein interaction with A3G and A3B by co-expression in culture cells. As multiple hnRNPs have been reported in A3 complexes (16, 23, 24), representative A1 cofactors including ACF, GRY-RBP, KSRP, and RBM47 and hnRNPs including hnRNP-A1, A2B1, D, E2, L, and R were chosen for investigating protein interactions with A3G by co-expression in human 293 FT and HepG2 cells. Their protein interactions with A3G were evaluated by immune-precipitation using an antibody against a FLAG tag incorporated in the N or C-terminal of these representative proteins. As shown in Fig. 1C, all A1 cofactors and hnRNPs tested had detectable protein interactions with A3G by co-expression in 293 FT cells. ACF, GRY-RBP, and KSRP are the major regulatory cofactors of A1, and their expression levels were lower or comparable with other hnRNPs tested. However, their protein interactions with A3G were significantly stronger than other hnRNPs in 293 FT cells. All these proteins had similar or lower expression in HepG2 cells. The protein interactions between A3G and the major A1 cofactors ACF, GRY-RBP, and KSRP were also detected in HepG2 cells. The data demonstrate that A1 cofactors have a preferential position in A3G complex under natural conditions.

The protein interactions observed above could be direct protein binding or indirect through binding to the same RNA. To further investigate A3 protein interactions with A1 cofactors or other hnRNPs in cellular conditions, A3B was transiently transfected in 293 FT cells with or without GRY-RBP or hnRNP-A1 co-expression. A3B protein interactions with endogenous or co-expressed GRY-RBP and hnRNP-A1 were evaluated by IP using an antibody against the FLAG tag in the C-terminal of A3B with or without RNase pre-treatment. As shown in Fig. 1D, the endogenous or co-expressed GRY-RBP and hnRNP-A1 were clearly detected in the A3B IPs. A3B protein interaction with GRY-RBP remained detectable after RNase pre-treatment. However, A3B protein interaction with hnRNP-A1 disappeared, indicating that the A3B and GRY-RBP interaction was direct protein binding, but A3B and hnRNP-A1 interaction was indirect through binding to the same RNA. Taken together, these data suggest that A1 cofactors and other RNPs are associated with A3 complexes directly or indirectly through protein or RNA interactions.

### A1 cofactors and other hnRNPs stimulate A3C mutational activity on HBV DNA

**Fig 2:**
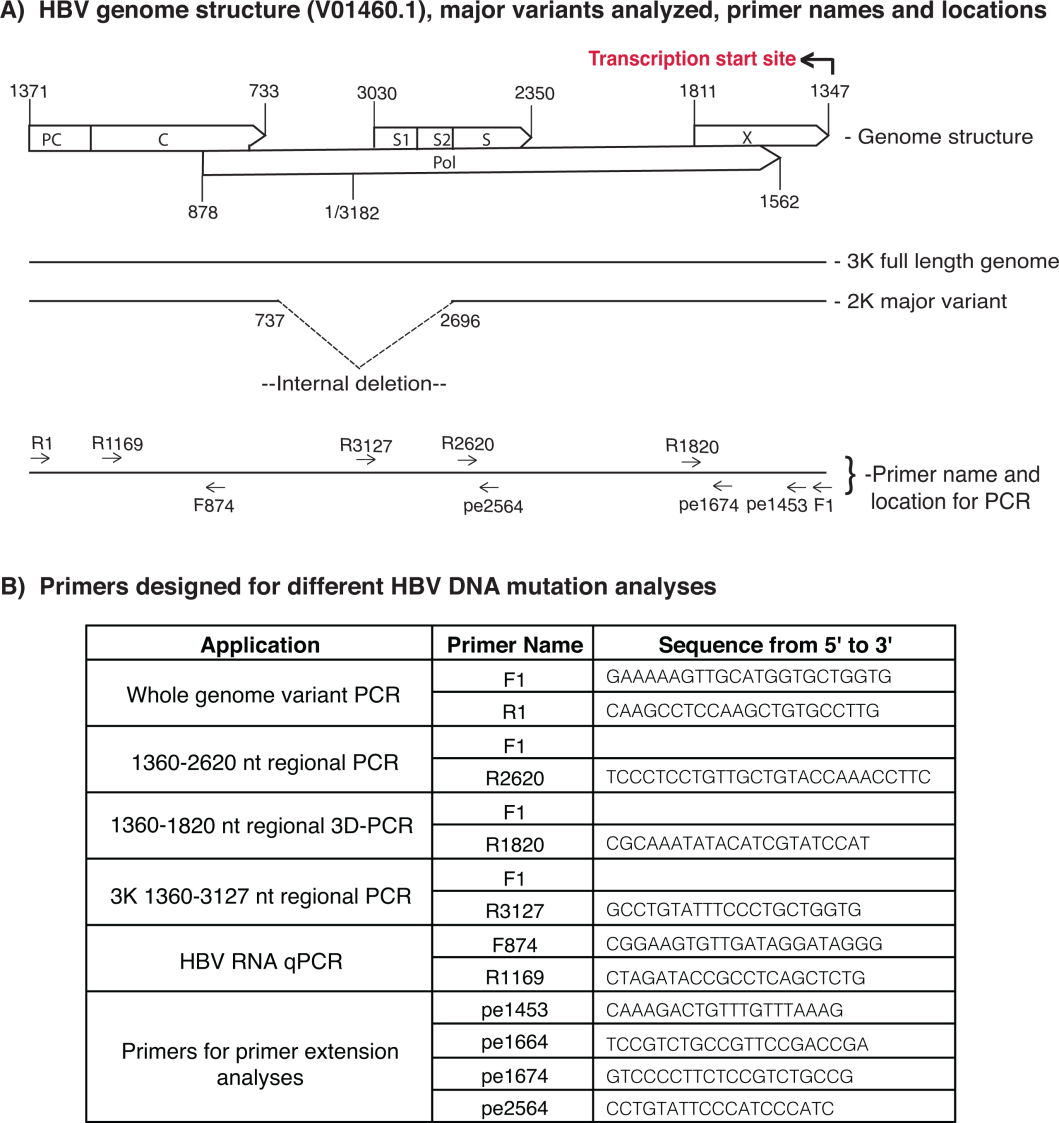
HBV genome variant structure and PCR designs for different applications. (A) The diagram of the *ayw* strain HBV genome structure with the reverse transcription starting site and major genome variants with internal deletion locations. Primer names and locations are presented in the lower panel. The nucleotide positions are numbered according to HBV V01460.1 in the gene bank. (B) HBV DNA PCR amplification designs for primer extension, sequencing analyses, or HBV RNA quantitation. Primers used for different HBV DNA PCR applications were listed with their locations and sequences.

To investigate the role of those cellular factors mentioned above on A3 mutation, A1 cofactors, and other representative hnRNPs were individually co-expressed with A3 and HBV encoding plasmids in HepG2 cells, a cellular HBV replication model mimicking HBV infection *in vivo* (14, 30). The resultant HBV viral rcDNAs were isolated, and HBV DNAs were amplified by PCR-95°C. The A3-induced mutational activities on HBV were evaluated by C-to-T mutational frequency analyses in the HBV PCR-95°C amplicons using the primer extension (pe) or by sequencing analyses through TA cloning of HBV PCR-95°C amplicons. HBV structure and regional or whole genome amplification designs are shown in Fig. 2. Different HBV DNA region amplifications were selected according to experimental target and HBV genome variants with internal deletion.

**Fig 3:**
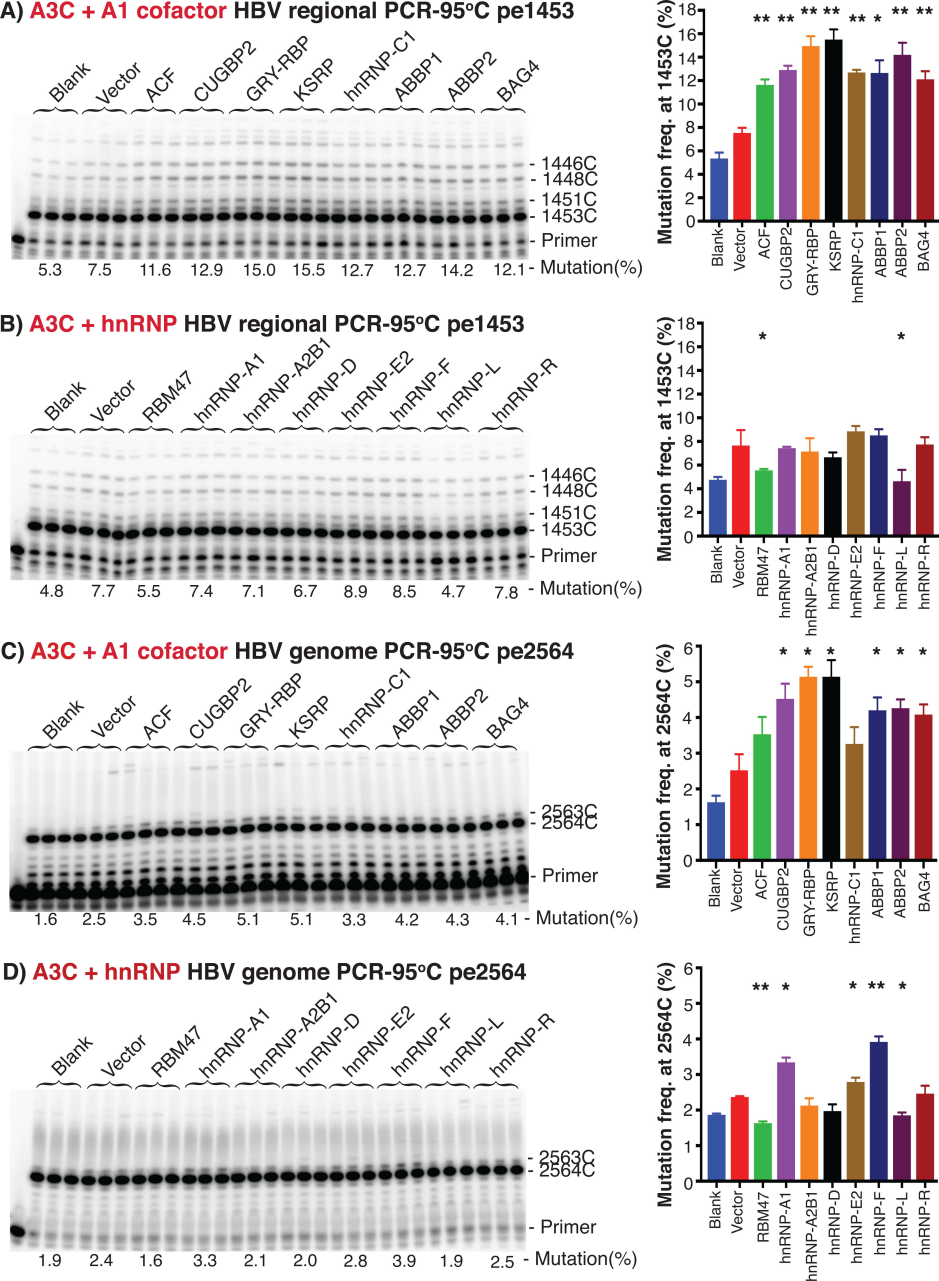
A1 cofactors and other hnRNPs stimulated A3C mutational activity by primer extension analyses. A1 cofactors or other representative hnRNPs were co-transfected with A3C and HBV encoding plasmids into HepG2 cells. The resultant HBV rcDNAs in the viral capsids were isolated and HBV genome variants’ common region 1360–2620 nt or whole genome variants were amplified by PCR-95°C. HBV C-to-T mutation frequencies at cytidine sites 1453 and 2564 in the PCR amplicons were determined by primer extension analyses, named pe1453 and pe2564, respectively. The ^32^P-labeled primer extension products were separated by an 8% polyacrylamide sequencing denaturing gel and quantitated by PhosphoImager. Mutation frequencies were determined by a percentage of these bands above the target cytosine site divided by the total extension product counts in the lane. The representative set data are presented as gel analyses of the primer extension products in the left panel and graph of the mutational frequencies in the right panel. (A) A1 cofactors’ effect and (B) other representative hnRNPs’ effect on A3C mutational activity by pe1453 using HBV 1360–2620 nt region amplicons. (C) A1 cofactors’ effect and (D) other representative hnRNPs’ effect on A3C mutational activity by pe2564 using HBV whole genome amplifications. Blank, background control by mock vector without A3C co-transfection. Vector, A3C alone control with mock vector to balance the total plasmid DNA amount for cell transfection. The “pe” stands for primer extension. Graph bar values are means + SD of 3 independent samples. *P < 0.05, **P < 0.001 indicate a statistically significant difference compared with the vector, A3C alone control.

As shown in Fig. 3A, A3C alone had a 7.5% C-to-T mutational frequency in contrast to the 5.3% blank background control due to endogenous A3C and A3G expressions (14), when utilizing PCR-95°C amplicons of the HBV region 1360–2620 nt by pe1453 analyses. Co-expression of A1 cofactors stimulated A3C mutational frequency from 7.5% up to 15.5% with variable effects by different cofactors. The major A1 cofactors, GRY-RBP and KSRP, had the largest stimulatory effect and increased A3C mutation frequency about 2-fold from 7.5% to 15.0% and 15.5%, respectively. All remaining A1 cofactors had variable stimulatory effects from 7.5% up to 14.2%. All gene over-expressions in HepG2 cells under the experimental conditions were confirmed by quantitative RT-PCR analyses (see Fig. S1). Although A1 cofactors had different over-expression levels, A3C co-expression levels were comparable except with ABBP1. In addition, the relative mRNA ratios between A3C and A1 cofactors were about 1:1–2.5, suggesting that there should be higher A1 cofactor levels than A3C levels for their combined effect on HBV DNA mutational activity. The stimulatory effect of A1 cofactors on A3C C-to-T mutational frequency together with their protein interaction with A3C demonstrate that A1 cofactors up-regulate A3C mutational activity.

Since other hnRNPs also interacted with A3s (16, 23, 24), hnRNP-A1, A2B1, D, E2, F, L, and R were representatively evaluated for their effect on A3C mutational activity by co-expression in the same way. As shown in Fig. 3B, the effect of these hnRNPs was smaller than that of A1 cofactors, but detectable. The co-expression of hnRNP-E2 and -F had detectable stimulatory effects on A3C from 7.7% up to 8.9% by pe1453 analyses with HBV 1360–2620 nt regional PCR-95°C amplificons. RBM47 (an essential component for A1 function like ACF) and hnRNP-L had inhibitory effects. However, others including hnRNP-A1, A2B1, D, and R had no significant effect on A3C mutational activity, indicating that these representative hnRNPs are less effective than A1 cofactors. Quantitative RT-PCR analyses also showed that hnRNPs were highly over-expressed, even higher than A1 cofactors after being normalized to the GAPDH housekeeping gene (see Fig. S2). The different effect of hnRNPs on A3C mutational activity reflected their different roles in the A3C complex.

The HBV viral genome has multiple variants with the major ones being the full-length 3K and the internal deletion 2K form due to alternative splicing as shown in Fig. 2. A3C-induced mutations varied depending on HBV genome variants (14). Therefore, HBV whole genome variants were amplified by PCR-95°C to further evaluate A3C mutational frequency using the primer extension analyses against 2564 nt site cytidine (pe2564). As shown in Fig. 3C, A1 cofactors had a stimulating effect on A3C mutation as determined by pe2564 analyses. The co-expression of GRY-RBP and KSRP stimulated A3C mutational activities about 2-fold from 2.5% up to 5.1% in contrast to the blank background control of 1.6%. Remaining A1 cofactors also stimulated A3C mutational activity with a pattern like those in Fig. 3A by pe1453 of HBV regional PCR amplicons. As shown in Fig. 3D, other representative RNPs also had detectable stimulatory or inhibitory effects by pe2564 analyses. HnRNP-A1 and F increased A3C mutational frequency from 2.4% up to 3.3% and 3.9%, respectively, with a larger change than those by pe1453 in Fig. 3B. Overall, A1 cofactors were still more effective than other hnRNPs by pe2564 of HBV whole genome amplicons, consistent with those by pe1453 analyses of HBV regional PCR amplicons. These data provide further evidence that A1 cofactors and other hnRNPs are potentially involved in the functional regulation of A3C mutation activity on HBV virus.

**Fig 4:**
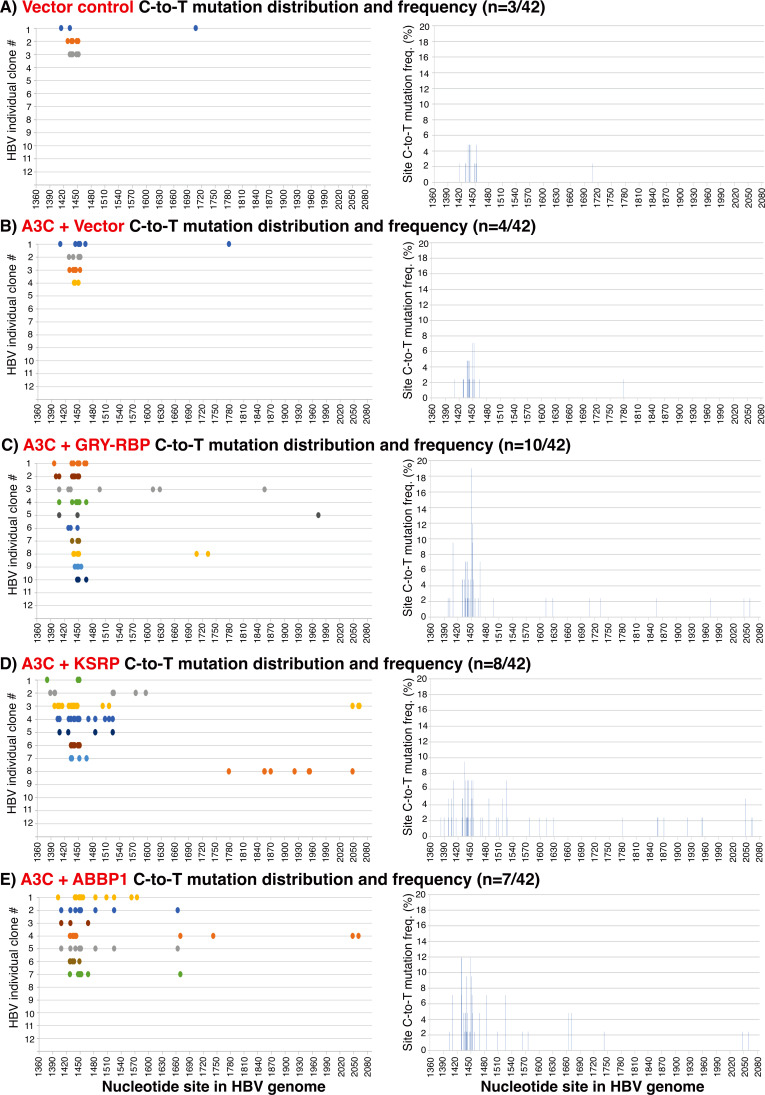
A1 cofactors increase A3C clonal mutation frequency and efficiency by sequencing analyses. Representative A1 cofactors, GRY-RBP, KSRP, and ABBP1, were co-transfected with A3C into HepG2 cells. After a 48 h transfection, HBV rcDNAs in the viral capsids were isolated, and HBV genomic variants’ common region 1360–2090 nt were amplified by PCR-95°C. The resultant amplicons were TA-cloned into a pCR4 vector for sequencing analyses. Forty-two clones were randomly selected for each treatment, and the clonal C-to-T mutation distributions against the corresponding cytidine site in the HBV V01460 genome are linearly presented as color spots along the line. The mutation positive clones for each treatment were also combined to evaluate the site mutational frequencies for each cytidine in the HBV region. The cytidine site mutational frequencies represent the number of C-to-T mutations for a specific cytidine site in the mutation positive clones divided by the sequenced clone number (*n* = 42) and are presented as a percentage bar against each cytidine site in the HBV PCR region. The C-to-T mutation distributions of mutation positive clones and their clonal combination site mutational frequencies are graphically presented for the treatment of (A) Vector control, (B) A3C + Vector, (C) A3C + GRY RBP, (D) A3C + KSRP, and (E) A3C + ABBP1.

Sequencing analysis is a conventional method to verify DNA mutation changes, although it is less sensitive than primer extension method above. To further investigate A1 cofactor effect on A3C, the HBV 1360–2090 nt regional PCR-95°C amplifications as in Fig. 3A were sequencing-analyzed. As shown in Fig. 4, co-expression of GRY-RBP, KSRP, and ABBP1 increased C-to-T mutation positive clones from 4 to 10, 8, and 7, respectively, in contrast to three in the vector background by 42 randomly screened clones. In addition, these A1 cofactors also increased C-to-T mutation numbers in each individual clone seen as more color spots along the clonal line. The C-to-T mutation distributions by A3C alone were mainly in a narrow region around 1450 nt. The A1 cofactor co-expression had broader C-to-T distribution in the region around 1450 nt, even across the whole PCR region as shown in Fig. 4D and E. In addition, the clonal combined C-to-T mutation frequency around 1450 nt increased from 7.1% by A3C alone to 19, 9.5, and 11.9% by the co-expression of GRY-RBP, KSRP, and ABBP1, respectively.

As summarized in Table 1 , compared with A3C alone, co-expression of GRY-RBP, KSRP, and ABBP1 increased the total C-to-T mutation number detected in 42 clones from 21 to 55, 68, and 60 with average C-to-T mutations per clone from 5.25 to 5.50, 8.50, and 8.57, respectively. Considering a total of 182 cytidines available in the HBV 1360–2090 nt region, clonal mutation efficiency can be defined as a percentage of the number of C-to-T mutations in a single mutation positive clone divided by the total 182 cytidines available in the clone. The co-expression of GRY-RBP, KSRP, and ABBP1 increased A3C clonal cytidine mutational efficiency from 2.88% to 3.02%, 4.67%, and 4.71%, respectively. Importantly, when the highest mutation clone under each treatment was taken into consideration, the co-expression of GRY-RBP, KSRP, and ABBP1 increased A3C clonal cytidine mutation efficiency from 3.30% to 4.95%, 10.99%, and 8.24%, respectively, indicating that up to a 3-fold hypermutation resulted from A1 cofactor co-expression. These data demonstrate that A1 cofactors not only increased A3C mutation frequency in HBV clonal population but also A3C mutation efficiency in individual molecular clone.

**TABLE 1:** Stimulating effect of A1 cofactors on A3C or A3G mutational activities by sequencing analyses of regional HBV PCR

HBV target	Treatment	Total sequenced clone number (n)	Mutation positive clone number (x/n)	C-to-T mutation # detected in each individual mutation positive clone	Total C-to-T # detected	Average C-to-T# per clone	Average C-to-T# /total C^*a*^ available per clone (%)	Clonal highest C-to-T # /total C^*a*^ available per clone (%)
1360–2090 nt PCR-95^o^C	Vector	42	3	6, 5, 3	14	4.67	2.57	3.30
	A3C + Vector	42	4	6, 6, 5, 4	21	5.25	2.88	3.30
	A3C + GRY RBP	42	10	9, 9, 7, 7, 6, 4, 4, 3, 3, 3	55	5.50	3.02	4.95
	A3C + KSRP	42	8	20, 15, 8, 7, 7, 4, 4, 3	68	8.50	4.67	10.99
	A3C + ABBP1	42	7	15, 12, 9, 9, 7, 5, 3	60	8.57	4.71	8.24
1360–1820 nt 3D-PCR-88^o^C	A3G + Vector	50	7	8, 7, 5, 5, 3, 3, 3	34	4.9	4.2	6.8
	A3G + CUGBP2	50	10	28, 18, 16, 10, 8, 5, 5, 4, 3, 2	99	9.9	8.5	23.9
	A3G + GRY RBP	50	11	18, 11, 8, 8, 7, 5, 4, 4, 3, 3, 3	74	6.7	5.7	15.4
	A3G + ABBP2	50	14	36, 23, 11, 10, 9, 8, 8, 7, 5, 5, 4, 3, 3	137	9.8	8.4	30.8

^*a*^ Total cytidines available for potential mutation per clone are 182 and 117 in the HBV regional amplicons of A3C PCR-95°C and A3G 3D-PCR-88°C, respectively. Mutational efficiency definition: a percentage of the number of C-to-T mutations in a single mutation positive clone divided by the total cytidines available in the clone.

### The A1 cofactor stimulating effect was also observed with other A3s

Compared with A3C, A3G, and A3B have been studied more extensively for their interactions with hnRNPs due to A3G’s antiviral effect on HIV-1 and A3B’s involvement in genomic DNA mutation in cancer (16, 23, 24). Therefore, we also evaluated the potential role of A1 cofactors and other representative hnRNPs on A3G and A3B mutational activities by primer extension and sequencing analyses. Briefly, A1 cofactors or other representative hnRNPs were co-expressed with A3G or A3B and HBV encoding plasmids in HepG2 cells as in Fig. 3. The resultant HBV rcDNAs were isolated, and the HBV 1360–2620 nt common region of all HBV genome variants were amplified by PCR-95°C for primer extension analyses.

**Fig 5:**
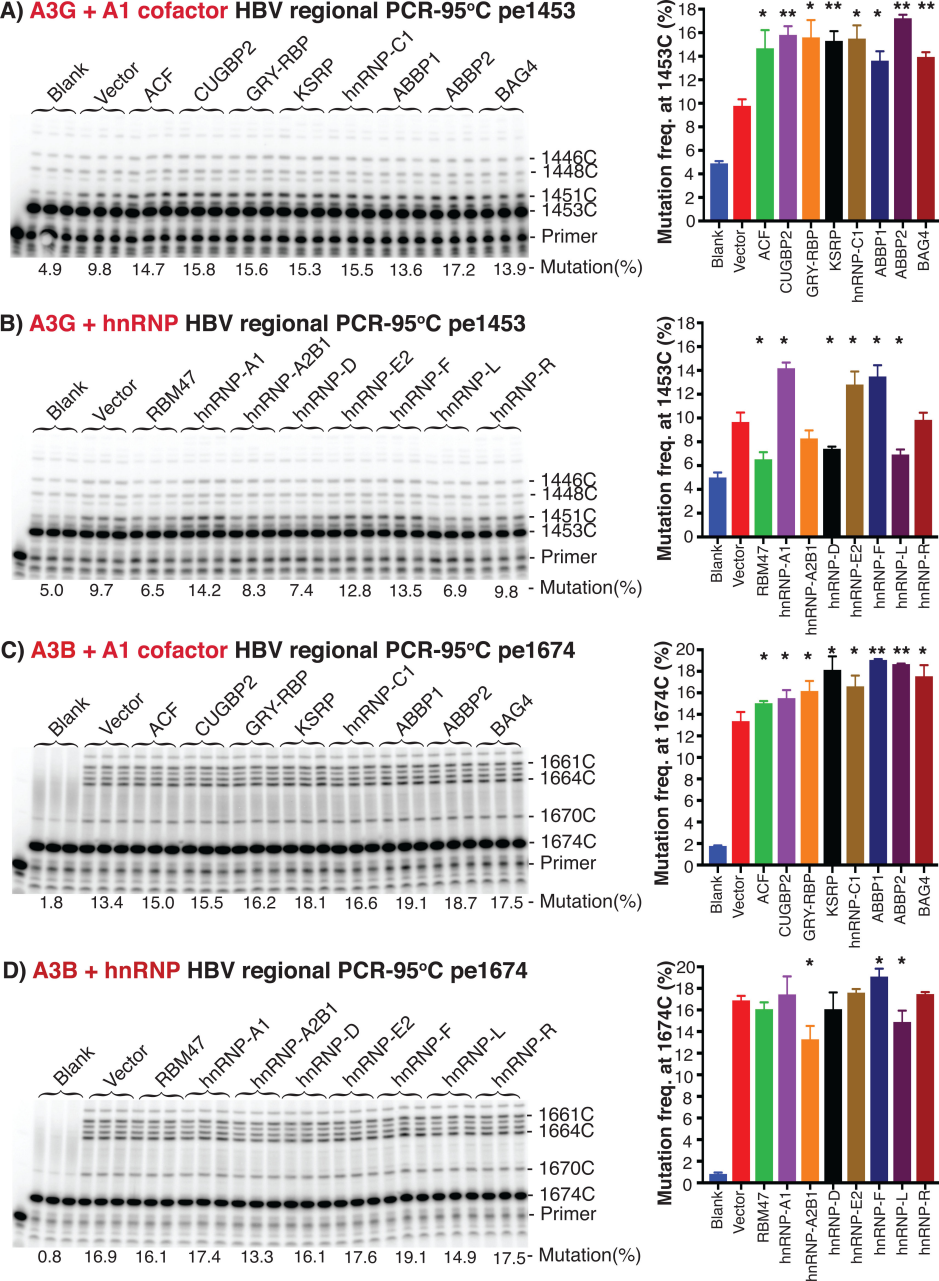
A1 cofactors and other hnRNPs stimulated A3G and A3B mutational activity as determined by primer extension analyses. A1 cofactors or other representative hnRNPs were co-transfected with A3G or A3B and HBV encoding plasmids into HepG2 cells. The resultant HBV rcDNAs in the viral capsids were isolated, and HBV genome variants’ common region 1360–2620 nt were amplified by PCR-95°C. After removal of dNTP, HBV C-to-T mutation frequencies at cytidine sites 1453 or 1674 nt in the HBV amplicons were determined by primer extension analyses, named pe1453 and pe1674 for A3G and A3B, respectively. The ^32^P-labeled primer extension products were separated by an 8% polyacrylamide sequencing denaturing gel followed by PhosphoImager quantitation for mutation frequencies as in Fig. 3. Representative data sets are presented with the primer extension product gel analyses in the left panel and a mutation frequency graph in the right panel. (A) A1 cofactors’ effect and (B) other representative hnRNPs’ effect on A3G mutational activity by pe1453. (C) A1 cofactors’ effect and (D) other representative hnRNPs’ effect on A3B mutational activity by pe1674. Blank*,* background control by mock vector without A3 co-transfection. Vector, A3G or A3B alone control with mock vector to balance the total plasmid DNA amount for the cell transfection. The “pe” stands for primer extension. Graph bar values are means + SD of 3 independent samples. *P < 0.05, **P < 0.001 indicate a statistically significant difference compared with the vector, A3G or A3B alone control.

As shown in Fig. 5A, all A1 cofactors stimulated A3G mutational activity by pe1453 analyses. A1 cofactor co-expression stimulated A3G mutation frequency from 9.8% by A3G alone to 13.6%–17.2% in contrast to the 4.9% blank background control. ABBP2 had the greatest (17.2%) stimulation on A3G, resulting in a 1.8-fold increase. GRY-RBP and KSRP also increased A3G mutational frequency like those in Fig. 3, indicating that A1 cofactors also regulate A3G mutational activity. As shown in Fig. 5B, the representative hnRNP co-expression also had a similar effect on A3G mutation like the A3C results in Fig. 3D. HnRNP-A1, E2, and F co-expression increased A3G mutation frequency from 9.7% by A3G alone up to 14.2%. On the other hand, RBM47 and hnRNP-L co-expression decreased A3G mutation from 9.7% down to 6.5%. Overall, A1 cofactors more effectively stimulated A3G induced mutation than the representative hnRNPs. All these gene over-expressions in HepG2 cells under the experimental conditions were confirmed by quantitative RT-PCR analyses (see Fig. S1 and S2). Although A1 cofactors or hnRNPs had variable over-expression levels, A3G co-expression levels were comparable, and the mRNA levels of A1 cofactors and hnRNPs were higher than that of A3G. In addition, over-expression of A1 cofactors or hnRNPs alone did not cause HBV DNA mutation, although they affected HBV viral replication levels. HBV DNA mutation was solely dependent on A3G presence (see Fig. S3). These data demonstrate that like those of A3C, both A1 cofactors and other hnRNPs had similar stimulation effects on A3G mutation. The higher stimulating effect of A1 cofactors than hnRNPs together with their greater protein interaction with A3G as shown in Fig. 1C indicate that A1 cofactors might have a unique regulatory role in A3-induced mutation.

A1 cofactor and other hnRNP effects on A3B were also investigated. A1 cofactor co-expression had a similar stimulatory effect on A3B mutation, but the increased magnitudes were much smaller than those of A3C or A3G. As shown in Fig. 5C, A1 cofactor co-expression increased A3B mutation frequencies from 13.4% by A3B alone up to 19.1% by pe1674, consistent with their effect on A3C and A3G above. However, the greatest increase by ABBP1 on A3B mutation was only 1.4-fold, which was much smaller than the 2.1-fold increase by KSRP on A3C or 1.8-fold increase by ABBP2 on A3G. As shown in Fig. 5D, the stimulatory effect of hnRNP-A1, E2, and F on A3B mutation was barely detectable, whereas an up to 1.4-fold increase was observed with A3G. Although the effect was weaker, A1 cofactors were overall more effective than other hnRNPs on A3B mutation, consistent with the results of A3C and A3G. The gene over-expression pattern of A1 cofactors or other hnRNPs was similar to those with A3C and A3G. A3B co-expression mRNA levels were comparable except with hnRNP-D and -L (see Fig. S1 and S2). These data demonstrate that A1 cofactors and other hnRNPs can regulate multiple A3 mutational activities. Considering the stimulatory magnitudes on different A3s, the data also indicate that A1 cofactors increase A3s’ mutational activity with an order of A3C > A3G>A3B.

**Fig 6:**
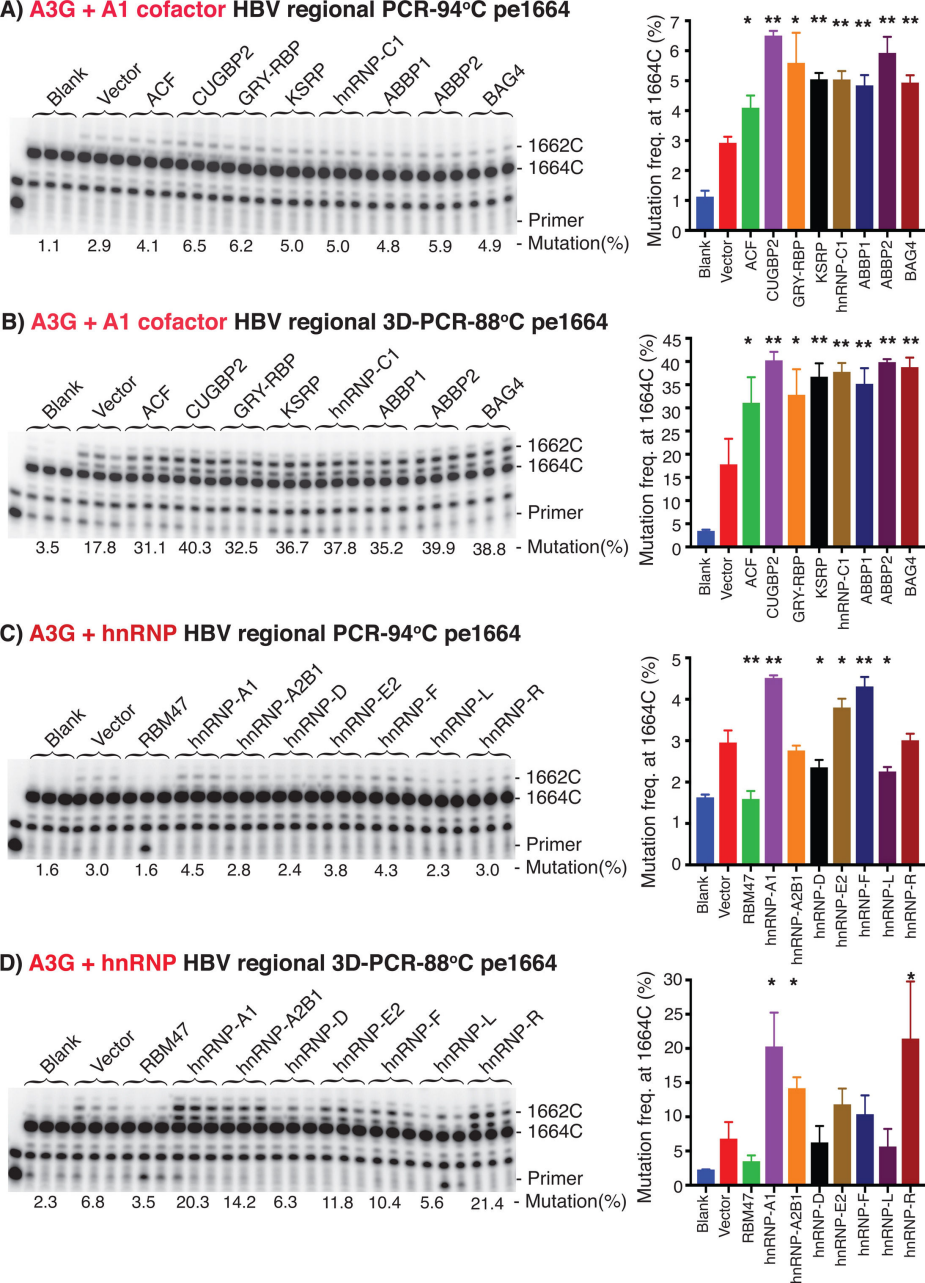
A1 cofactors and other hnRNPs stimulate A3G mutational activity as determined by analyses of 3D-PCR followed by primer extension. A3G and HBV encoding plasmids were co-transfected with A1 cofactors or other representative hnRNPs into HepG2 cells. HBV rcDNAs were isolated from the viral capsids, and HBV DNA 1360–1820 nt region was amplified by PCR-94°C. The potential mutants in the PCR-94°C were further enriched by 3D-PCR-88°C. After removal of dNTP, both PCR-94°C and 3D-PCR-88°C amplicons were used to determine HBV C-to-T mutational frequencies at cytidine site 1664 nt by pe1664. The data are presented with the primer extension product gel analyses in the left panel and the mutational frequency graphs in the right panel for the treatment of (A) and (B) A1 cofactors or (C) and (D) other representative hnRNPs on PCR-94°C and 3D-PCR-88°C, respectively. Triplicate samples were used for each determination. Graph bar values are means + SD of three independent samples. *P < 0.05, **P < 0.001 indicate a statistically significant difference compared with the vector, A3G alone control.

The C-to-T mutations induced by A3s result in AT-rich DNAs that have a lower denaturing temperature for PCR and can be selectively amplified by differential DNA denaturation-PCR (3D-PCR) (31). 3D-PCR analyses were often used for viral DNA mutation determinations including HBV. Utilizing 3D-PCR, we further investigated the stimulating effect of A1 cofactors and other hnRNPs on A3G C-to-T mutation frequency and efficiency by 3D-PCR-88°C amplifications through the primer extension and sequencing analyses. As shown in Fig. 6A and B, A1 cofactors increased A3G mutation frequencies from 2.9% by A3G alone up to 6.2% in PCR-94°C by pe1664 analyses. However, A1 cofactors increased A3G mutational frequencies from 17.8% up to 40.3% by 3D-PCR-88°C, indicating that A3G C-to-T mutations were enriched in the 3D-PCR-88°C. Similarly, the increased effect of hnRNP-A1, A2B1, E, F, and R on A3G mutation became more significant in 3D-PCR-88°C than PCR-94°C as shown in Fig. 6C and D. These data provided further evidence that A1 cofactors and other representative hnRNPs did have significant stimulatory effect on A3G-induced C-to-T mutational activity.

**Fig 7:**
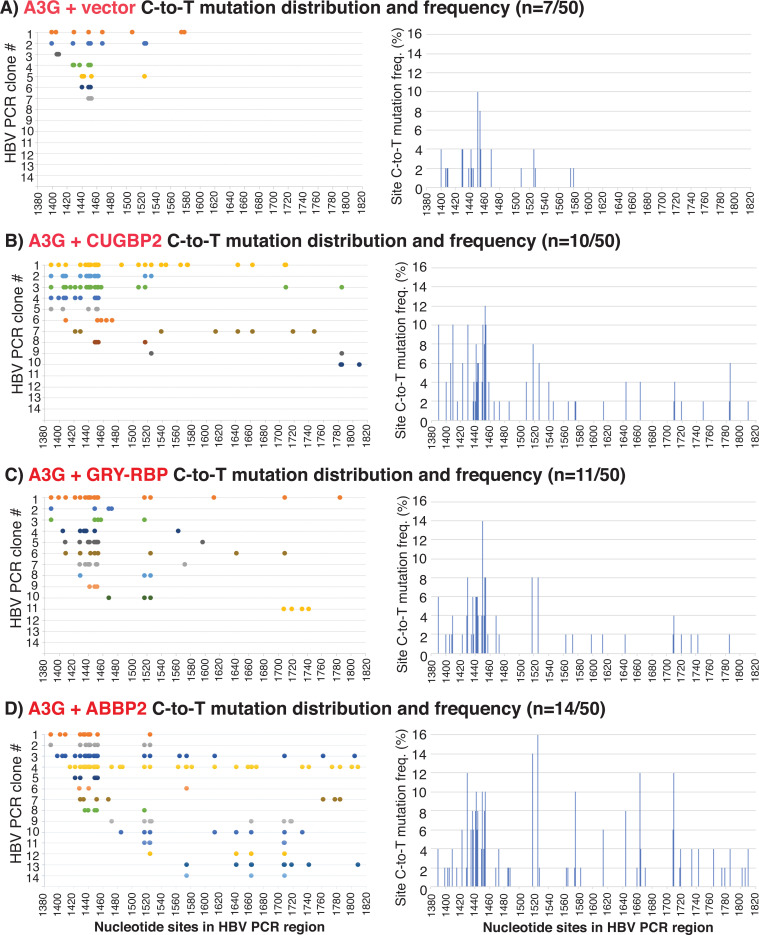
A1 cofactors increase A3G clonal mutational frequency and efficiency by sequencing analyses of 3D-PCR-88°C amplicons. A3G and HBV encoding plasmids were co-transfected with mock vector, CUGBP2, GRY-RBP, or ABBP2 into HepG2 cells. HBV rcDNAs were isolated, and HBV DNA 1360–1820 nt region was amplified by PCR-94°C followed by 3D-PCR-88°C as in Fig. 6. The resultant 3D-PCR-88°C amplicons were TA-cloned into a pCR4 vector. Fifty clones were randomly selected and analyzed by sequencing for the treatment of (A) A3G alone (A3G + Vector), (B) A3G + CUGBP2, (C) A3G + GRY RBP, and (D) A3G + ABBP2. The clonal C-to-T mutational distributions in mutation-positive clones for each treatment are linearly presented as color spots against the corresponding cytidine site in the HBV genome with site numbering reference to HBV V01460 in the left panel. The site cytidine mutational frequencies represent the number of C-to-T mutations for a specific cytidine site in the mutation-positive clones divided by the sequenced clone number (*n* = 50) and are presented as percentage bars for each cytidine site in the right panel.

The A1 cofactor effects on A3G in 3D-PCR-88°C were further verified by sequencing analyses. As shown in Table 1, co-expression of CUGBP2, GRY-RBP, and ABBP2 not only increased A3G mutation positive clone number from seven by A3G alone to 10, 11, and 14, respectively, by 50 randomly screened clones but also increased A3G mutation efficiency from average 4.2% by A3G alone up to 8.5%, 5.7%, and 8.4%, respectively. As shown in Fig. 7, these A1 cofactors not only increased A3G C-to-T mutation density for cytidines around 1450 nt but also induced more C-to-T mutations across the HBV PCR region, especially by ABBP2 as seen in Fig. 7D. These data confirm that A1 cofactors not only increased A3G mutation frequency in HBV clonal population but also increased A3G mutation efficiency in individual molecular clones, consistent with the results of A3C as in Fig. 4.

### Endogenous A1 cofactors and hnRNPs are actually involved in A3 mutational activity regulation

As described above, co-expression of A1 cofactors and other hnRNPs regulate A3 mutational activity on HBV virus in HepG2 cells. However, the actual protein components of A3 complexes under physiological conditions are unknown, and how these cellular factors interact with A3s during A3-induced mutation remains to be elucidated. To assess the involvement of these proteins under natural conditions, we performed representative siRNA knockdown of endogenous proteins in HepG2 cells including CUGBP2, GRY-RBP, ABBP1, hnRNP-A1, and hnRNP-L to evaluate their cellular effect on A3G- and A3C-induced HBV mutational activities.

**Fig 8:**
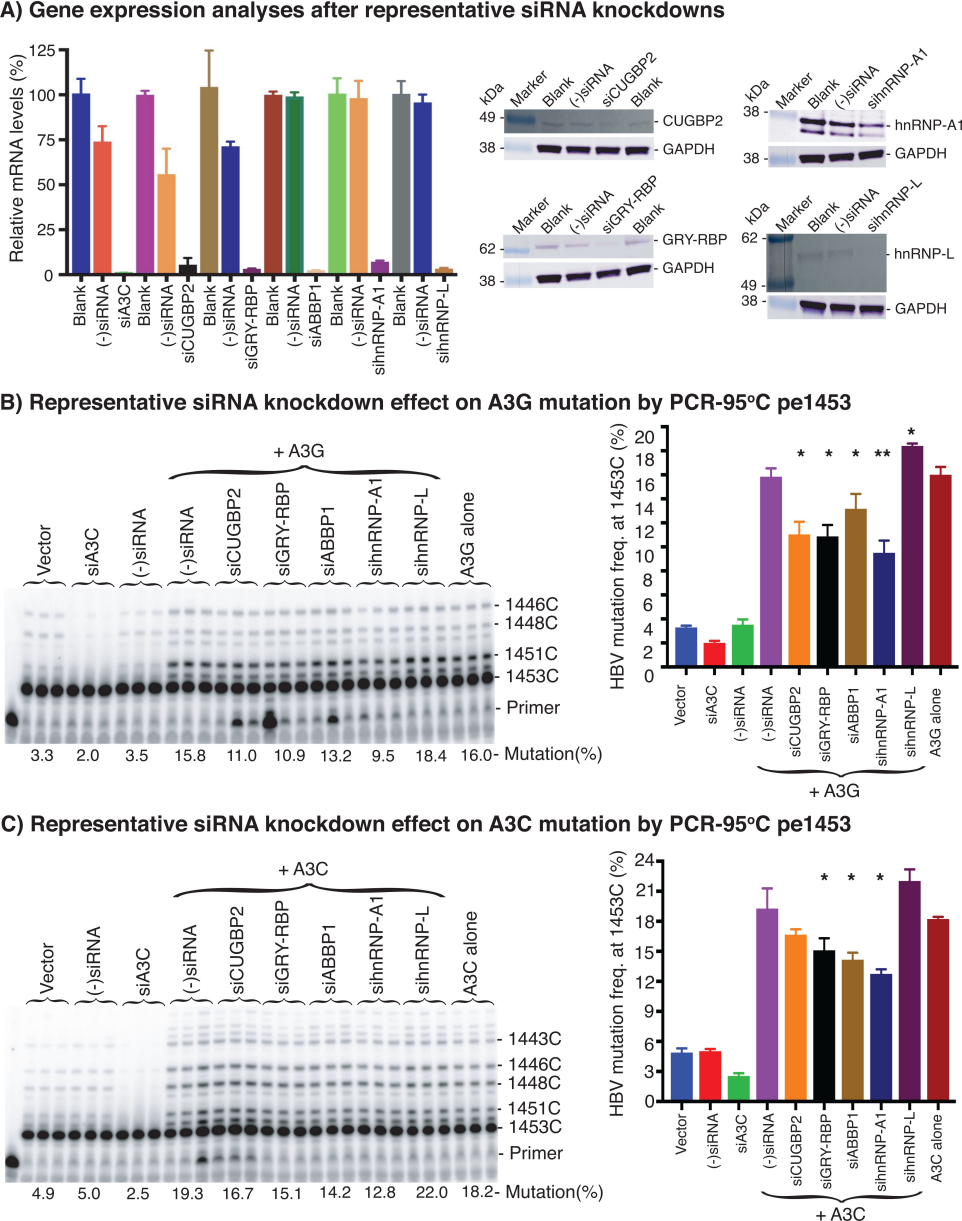
The siRNA knockdown effect of representative endogenous A1 cofactors and other hnRNPs on A3 mutational activities. Representative A1 cofactor or hnRNP siRNAs were transfected into HepG2 cells. After a 24 h siRNA treatment, the cells were transfected with A3G or A3C and HBV encoding plasmids. After a 48 h A3G or A3C transfection, the cells were harvested for HBV rcDNA or protein extractions. (A) Representative A1 cofactor or hnRNP mRNA and protein expression level analyses after siRNA treatment. The mRNA expression levels were determined by quantitative RT-PCR, and the data are represented as percentages relative to the blank and (−)siRNA control in the left panel. Triplicate samples were used for each determination. Representative protein expression levels were analyzed by western blotting, and the data are presented in the right panel. (B and C) Representative siRNA knockdown effect on A3G and A3C mutational activity analyses, respectively. HBV genome variants’ common region 1360–2620 nt were amplified by PCR-95°C. The siRNA knockdown effect on A3G or A3C mutation activity was determined by pe1453 in the resultant PCR amplicons. The data are presented with the primer extension product gel analyses in the left panel and a mutational frequency graph in the right panel. Vector, background control by mock vector without A3 co-transfection. (−)siRNA, another control by the scrambled negative siRNA with or without A3 co-transfection. Graph bar values are means + SD of 3 independent samples. *P < 0.05, **P < 0.001 indicate a statistically significant difference compared with the (−)siRNA + A3G or A3C.

As shown in Fig. 8A, siRNA treatments significantly reduced endogenous mRNA expression levels of A3C, CUGBP2, GRY-RBP, ABBP1, hnRNP-A1, and hnRNP-L by >90%. Representative western blotting also demonstrated that siRNA knockdown significantly reduced the endogenous protein expression levels of CUGBP2, GRY-RBP, hnRNP-A1, and hnRNP-L. As shown in Fig. 8B and C, A3C siRNA knockdown effectively eliminated the background mutation level detectable by pe1453, consistent with the previous report that A3C is the major endogenous contributor to mutations in HepG2 cells (14).

As shown in Fig. 8B, the siRNA knockdown of CUGBP2, GRY-RBP, ABBP1, and hnRNP-A1 decreased A3G mutational frequency from 15.8% to 11.0%, 10.9%, 13.2%, and 9.5%, respectively, opposite to their over-expression stimulating effect shown in Fig. 5. On the other hand, the siRNA knockdown of hnRNP-L increased A3G mutation activity from 15.8% to 18.4%, consistent with the inhibitory effect by hnRNP-L co-expression seen in Fig. 5. These data demonstrated that endogenous CUGBP2, GRY-RBP, ABBP1, hnRNP-A1, and hnRNP-L were truly involved in regulating A3G mutations on HBV viral DNA. Similar siRNA knockdown effects were also observed with A3C. As shown in Fig. 8C, the siRNA knockdown of CUGBP2, GRY-RBP, ABBP1, and hnRNP-A1 also had small but consistent inhibitory effects from 19.3% down to 12.8% on A3C mutational activity, whereas hnRNP-L siRNA knockdown increased A3C mutation from 19.3% to 22.0%, like those with A3G. These data suggest that A1 cofactors and other hnRNPs are not only associated with A3 complexes but also play regulatory roles in A3 mutational activity under physiological conditions. In addition, small but consistent changes by siRNA knockdown and varied effects by co-expression also indicate that A3s are complex with multiple components that interact with each other dynamically to regulate A3 mutational activity.

### Disrupting A3G interactions with hnRNPs or HBV polymerase significantly decreases A3G mutational activity

As described above, endogenous A1 cofactors and other hnRNPs are involved in regulating A3 mutational activity. To further evaluate the role of these A3 complex associated proteins, we investigated the consequence of A3 mutagenesis that disrupted A3G or A3B protein interactions with hnRNPs or HBV viral polymerase. A3G and A3B have two deaminase domains. Their N-terminal deaminase domains are involved in interactions with RNA or hnRNPs to form high-molecular weight (HMW) complexes (16, 23, 24). A3G W94 and W127 are the major sites involved in A3G HMW complex assembly and RNA binding (23). Mutating A3G W94A or W127A impedes RNA binding and abrogates its RNA-dependent protein oligomerization while preserving A3G deaminase activity *in vitro*. On the other hand, A3G L35 is its protein interaction site with HIV-1 Polymerase (32). Therefore, we generated A3G L35A, W94A, and W94A + W127A mutants to investigate the effect of disrupting protein interactions on A3G mutational activity.

**Fig 9:**
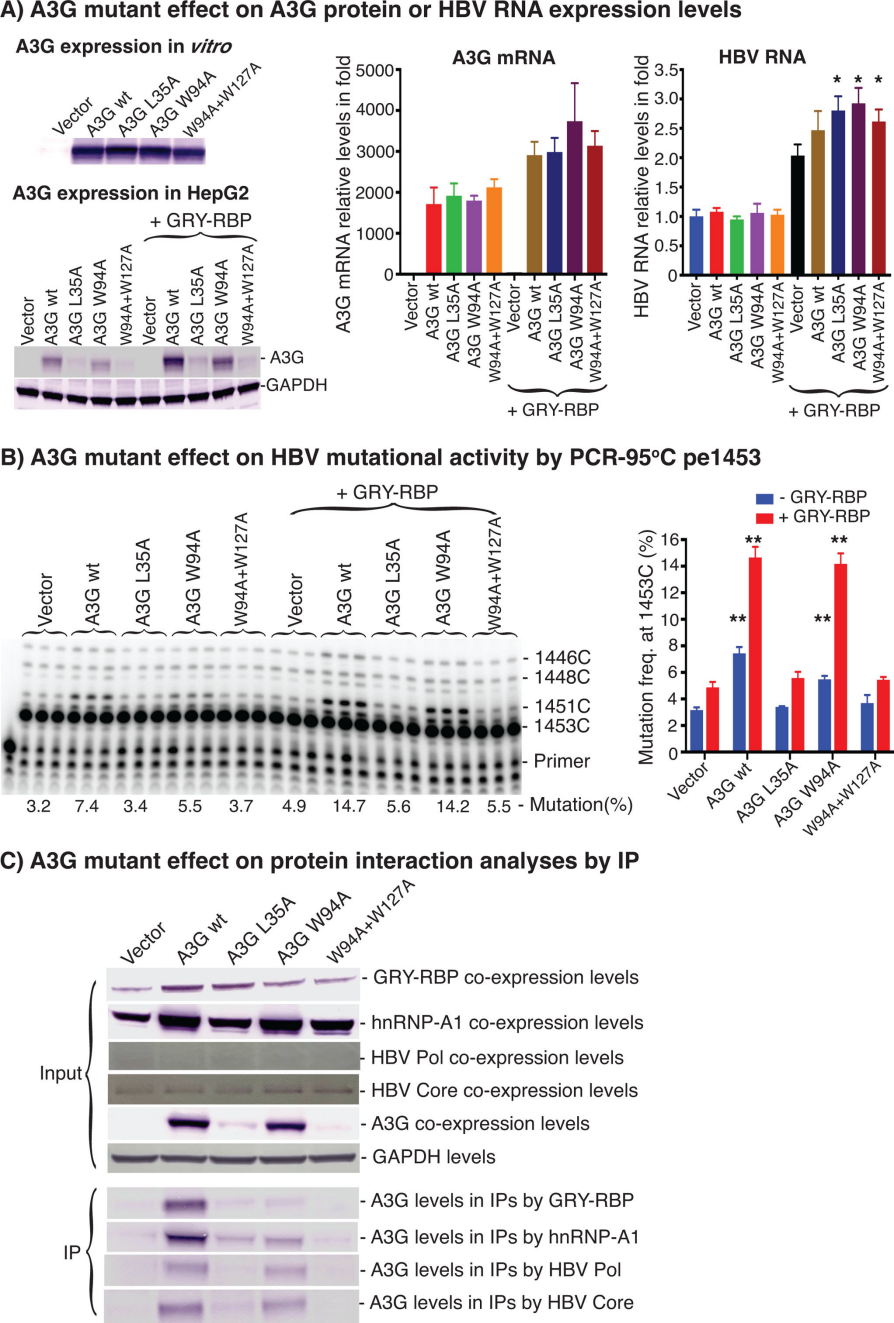
A3G protein stability and mutational activity are significantly inhibited by disrupting its protein interaction with hnRNPs or HBV Polymerase. A3G or its mutant encoding plasmids were co-transfected with or without GRY-RBP into HepG2 cells. After 48 h transfection, the cells were lysed for protein analyses or for HBV viral RNA or rcDNA isolations. (**A**) A3G mutagenesis effect on A3G protein or mRNA expression level analyses. A3G wild-type and its mutants were expressed by an *in vitro* coupled transcription/translation system or transfection in HepG2 cells. The *in vitro* or cellular A3G protein expression levels were analyzed by 10% polyacrylamide gel electrophoresis followed by western blotting. The data are presented in the left panel. A3G mRNA and HBV viral RNA expression levels were quantitated by RT-PCR, and the data are presented in the right panel. Graph bar values are means + SD of three independent samples. *P < 0.05 indicates a statistically significant difference compared with the Vector + GRYRBP. (**B**) A3G mutagenesis effect on HBV mutational activity analyses in HepG2 cells. HBV genome variants’ common region 1360–2620 nt were amplified by PCR-95°C. A3G mutagenesis effect on A3G mutation activity was determined by pe1453 in the PCR amplicons. The data are presented with the primer extension product gel analyses in the left panel and a mutational frequency graph in the right panel. Graph bar values are means + SD of three independent samples. *P < 0.05, **P < 0.001 indicate a statistically significant difference compared with the vector or vector + GRYRBP. (**C**) A3G mutagenesis effect on protein interaction analyses in 293 FT cells. A3G or its mutant encoding plasmids were co-transfected with GRY-RBP, hnRNP-A1, HBV Polymerase, or HBV Core protein into 293 FT cells. After a 48 h transfection, the cells were lysed, and the expression levels of GRY-RBP, hnRNP-A1, or A3G and its mutants were analyzed by western blotting. In parallel, A3G complexes in cell lysates were immune-precipitated (IP) by an antibody against the FLAG tag in the C-terminal of GRY-RBP, hnRNP-A1, HBV Polymerase, or HBV Core protein. A3G and its mutant levels in the IPs were analyzed by western blotting using an antibody against the HA tag in A3G and its mutants.

As shown in Fig. 9A, when A3G wild-type, L35A, W94A, and W94A + W127A were expressed by *in vitro* TNT quick-coupled transcription/translation systems, their *in vitro* protein expression levels were comparable. However, when they were expressed in HepG2 cells by transfection, their protein expression levels varied significantly. A3G W94A had levels comparable with A3G wild-type. However, A3G L35A and W94A + W127A levels were significantly decreased. Co-expression of GRY-RBP increased the expression levels of all A3Gs including both wild-type and mutants. These data suggest that A3G mutant stability is compromised when A3G protein interactions with RNAs or hnRNPs are disrupted. To rule out if A3G mutant protein level variations were potentially due to unequal transfection efficiency, A3G mRNA and HBV pgRNA were quantified by RT-PCR in the co-transfected HepG2 cells. As shown in the right panel of Fig. 9A, A3G mRNA and HBV pgRNA were readily detectable and comparable, indicating that the HBV constructs were replication competent, and the co-transfection model was not hampered by unequal transfection efficiency.

As shown in Fig. 9B, all A3G mutants significantly inhibited A3G mutational activity on HBV DNA by pe1453 analyses. A3G wild-type had a 7.4% C-to-T mutational frequency. A3G L35A and W94A + W127A mutants decreased A3G mutational activity from 7.4% to 3.4% and 3.7%, respectively, a level almost identical to the 3.2% background control, indicating almost total loss of A3G mutational activity. On the other hand, GRY-RBP co-expression increased A3G wild-type mutational frequency from 7.4% to 14.7% as well as A3G W94A mutant from 5.5% to 14.2%, indicating that GRY-RBP has a significant compensatory effect on restoring A3 mutational activity. However, GRY-RBP co-expression only slightly increased the mutational activity of A3G L35A and W94A + W127A whereas their protein expressions were clearly detected, indicating significant loss of the mutant HBV mutational activity. These data suggest that disrupting A3G protein interactions with cellular hnRNPs not only compromised A3G protein stability but also significantly inhibited A3G mutational activity on HBV DNA. A3G protein interactions with cellular hnRNPs are essential for its mutational activity. Of note, A3G protein expression levels decreased significantly due to A3G mutagenesis, and the decreased protein levels could explain the lower A3G mutational activity on HBV. On the other hand, the L35A and W94A + W127A mutant proteins were clearly detectable as shown in Fig. 9A, especially with GRY-RBP co-expression, but their HBV DNA mutation levels were similar to the background control, indicating that A3G mutant enzyme activity was lost. Therefore, decreased A3G DNA mutational activity likely reflects contributions from both of these two effects and demonstrates that A3G requires cellular cofactors to be fully functional.

To verify if the A3G mutant activity changes above were associated with disrupted protein interactions, A3G mutants were co-expressed with C-terminal Flag-tagged GRY-RBP, hnRNP-A1, HBV Polymerase, and HBV Core protein in 293 FT cells. The protein interactions between A1 cofactors or other hnRNPs and A3G in HepG2 cells were comparable with that in 293 FT cell, but 293 FT cells were chosen for the relevant evaluation due to higher protein expression than in HepG2 (see Fig. 1C). The use of two cell lines instead of just a single one is a limitation for data interpretation. Their protein interactions were evaluated by immune-precipitation (IP) using an antibody against the Flag tags. As shown in Fig. 9C, the protein expression levels of GRY-RBP, hnRNP-A1, HBV Polymerase, and HBV Core were comparable with co-expression of different A3G mutants. However, A3G mutant protein expression levels varied, consistent with their expression seen in HepG2 cells in Fig. 9A. After immunoprecipitation, wild-type A3G was readily detected in all IPs pulled-down by GRY-RBP, hnRNP-A1, HBV Polymerase, or HBV Core protein, indicating a strong A3G protein interaction with all of these proteins. However, A3G levels decreased significantly in the IPs of the A3G mutants L35A, W94A, and W94A + W127A, indicating that the protein interactions were significantly disrupted by A3G mutagenesis. In addition, A3G levels were relatively higher in the IPs of L35A and W94A than W94A + W127A, indicating a higher A3G complex disruption by W94A + W127A, consistent with previous reports (23).

HBV Core and Polymerase co-expression protein levels were lower than GRY-RBP and hnRNP-A1 in the cell lysates by western blotting. However, A3G and its mutants were still detectable in their IPs, indicating the presence of A3G interactions with both HBV polymerase and core protein. As shown in Fig. 9C lower panel, A3G mutants in IPs by HBV core protein had levels parallel to their inputs. However, the amount of A3G L35A in the IP by HBV polymerase was less than that in the IP by HBV core protein, indicating that A3G L35A disrupted A3G protein interaction with HBV polymerase like HIV-1 polymerase. These data suggest that the significant inhibitory effect of the L35A mutant on A3G mutational activity is associated with decreased protein interactions with both GRY-RBP/hnRNP-A1 and HBV polymerase.

### A3B interaction with hnRNPs is essential for its HBV mutational activity

It has been reported that the A3G HMW complex could be dissociated completely by RNase treatment, but the A3B HMW complex cannot be dissociated (24), indicating that A3B has much stronger protein interactions with hnRNPs than A3G. Y to A mutations of the A3B tyrosine residues at 13, 28, 83, and 162 with or without W127A result in disruption of the A3B HMW complex formation (24). To investigate the contribution of hnRNPs to A3B, we evaluated A3B mutational activity by mutagenesis including W94A + W127A, Y13A + Y28A + Y83A + Y162A (4Y/A), 4Y/A + W94A, and 4Y/A + W127A.

**Fig 10:**
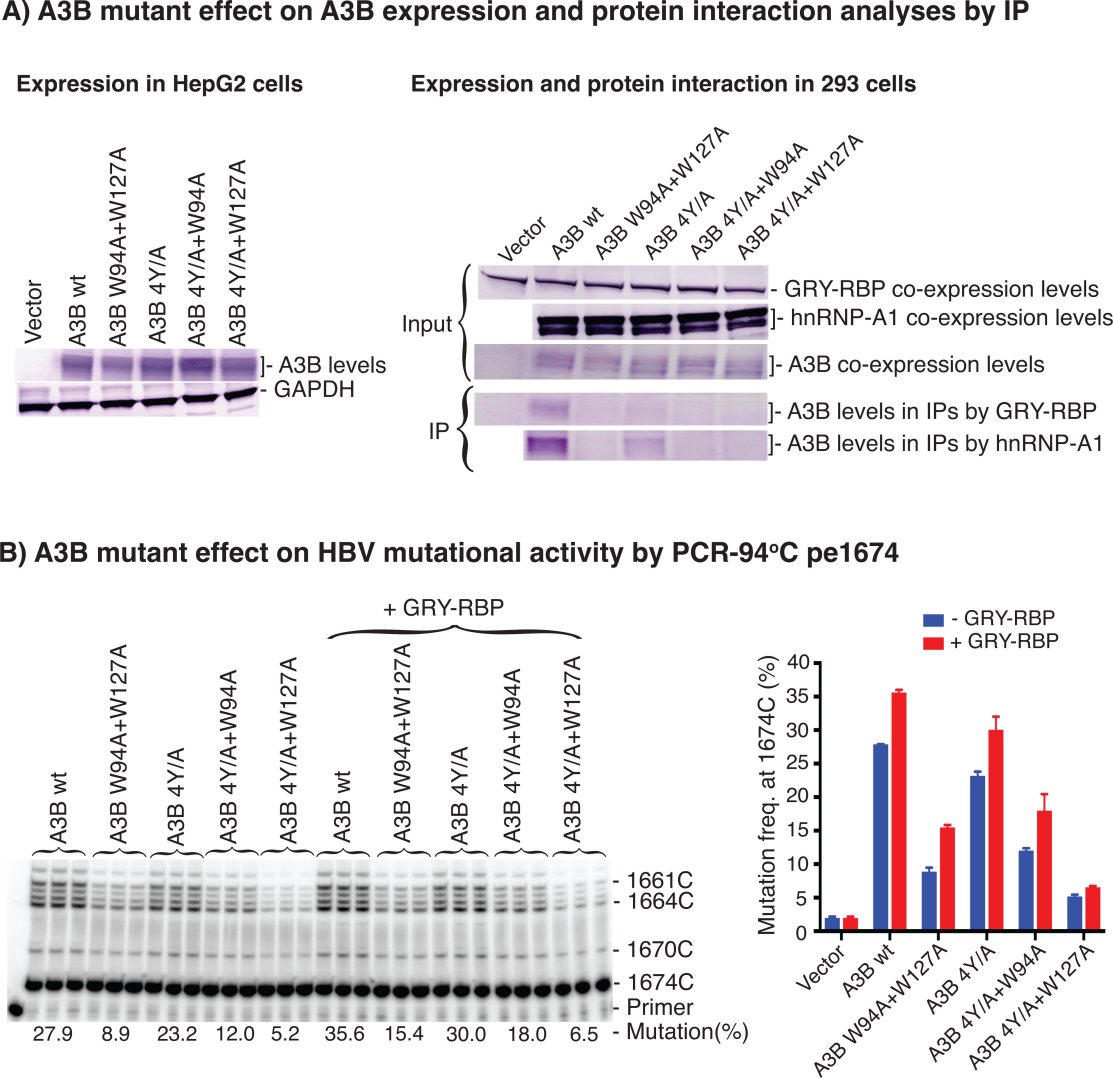
A3B mutational activity is significantly inhibited by disrupting its protein interaction with hnRNPs. (**A**) A3B mutagenesis effect on A3B protein expression level and protein interaction analyses. A3B wild-type and mutant encoding plasmids were transfected into HepG2 cells. A3B wild-type and mutant protein expression levels in HepG2 cells were analyzed by western blotting using an antibody against A3B. The data are presented in the left panel. Alternatively, A3B wild-type and mutant encoding plasmids were co-transfected with GRY-RBP and hnRNP-A1 into 293 FT cells. After a 48 h transfection, the cells were lysed, and the expression levels of GRY-RBP, hnRNP-A1, or A3B and its mutants were analyzed by western blotting. In parallel, A3B wild-type and mutant complexes in the cell lysates were immune-precipitated (IP) by an antibody against the FLAG tag in C-terminal of GRY-RBP and hnRNP-A1. A3B wild-type and mutant levels in the IPs were analyzed by western blotting using an antibody against A3B. The data are presented in the right panel. (**B**) A3B mutagenesis effect on HBV mutational activity analyses. A3B wild-type and mutant encoding plasmids were co-transfected with or without GRY-RBP into HepG2 cells. After a 48 h transfection, the resultant HBV rcDNAs were isolated, and HBV genome variants’ common region 1360–1820 nt were amplified by PCR-94°C. The A3B mutagenesis effects on A3B mutation activity were determined by pe1674 in the resultant PCR amplicons. The data are presented with the primer extension product gel analyses in the left panel and a mutational frequency graph in the right panel. Graph bar values are means + SD of three independent samples.

As shown in Fig. 10A, A3B mutants and A3B wild-type had comparable protein expression levels in HepG2 cells regardless of their site mutations, in contrast to the A3G mutagenesis results in Fig. 9 where A3G mutant protein expression levels decreased significantly. To verify if these A3B mutants had disrupted protein interactions with hnRNPs, A3B mutant protein interactions with GRY-RBP and hnRNP-A1 were investigated by IPs after their co-expression in 293 FT cells. As shown in Fig. 10A right panel, the input levels of GRY-RBP, hnRNP-A1, and A3B mutants were comparable. A3B wild-type was readily detectable in the IPs by antibodies against GRY-RBP or hnRNP-A1. However, A3B protein levels in the IPs of both GRY-RBP and hnRNP-A1 were significantly decreased by the 4Y/A mutant and were almost undetectable with the W94AW127A, 4Y/A + W94A, and 4Y/A + W127A mutants, indicating a significant disruption of the protein interactions between them.

As shown in Fig. 10B, A3B 4Y/A mildly decreased A3B mutational activity from 27.9% by A3B wt to 23.2% by pe1674 analyses. The A3B 4Y/A + W127A mutant significantly decreased A3B mutational activity from 27.9% to 5.2%, indicating that the protein interaction disruption by 4Y/A + W127A resulted in a significant decrease in A3B mutational activity. Similarly, 4Y/A + W94A and W94A + W127A mutants also significantly decreased A3B mutational activity from 27.9% to 12.0% and 8.9%, respectively, in association with their protein interaction disruption. On the other hand, GRY-RBP co-expression increased A3B wild-type activity from 27.9% to 35.6%. GRY-RBP co-expression also stimulated activities of all A3B mutants with increases paralleling that of the A3B wild-type except for the 4Y/A + W127A mutant. The 4Y/A + W127A mutant activity was only slightly increased from 5.2% to 6.5% by GRY-RBP co-expression, indicating that the lost A3B mutational activity by 4Y/A + W127A protein interaction disruption could not be restored by GRY-RBP. These data demonstrate that A3B protein interactions with hnRNPs in the complex were significantly disrupted by A3B mutagenesis at sites W94A, W127A, and 4Y/A and that these protein interaction disruptions were associated with decreased or even loss of A3B mutant activity, indicating that A3B interactions with hnRNPs are essential for its HBV mutational activity.

### GRY-RBP dramatically increases A3C accessibility to HBV (−)DNA and mutation efficiency to generate hypermutation by HBV genome sequencing analyses

A3C induced-HBV mutations occur on HBV (−)DNA during viral reverse transcription in a sequential manner with significantly varied C-to-T mutation efficiency (14). The stimulatory effect of A1 cofactors and other hnRNPs on A3C mutational activities as described above indicates that they are the cellular factors contributing to A3C mutation efficiency regulation. To investigate the potential mechanism, GRY-RBP and KSRP were representatively co-expressed with A3C and HBV encoding plasmids in HepG2 cells. The resultant HBV 2K and 3K genome variants were amplified by PCR-95°C, and their genome-wide mutation landscapes were analyzed by sequencing.

**Fig 11:**
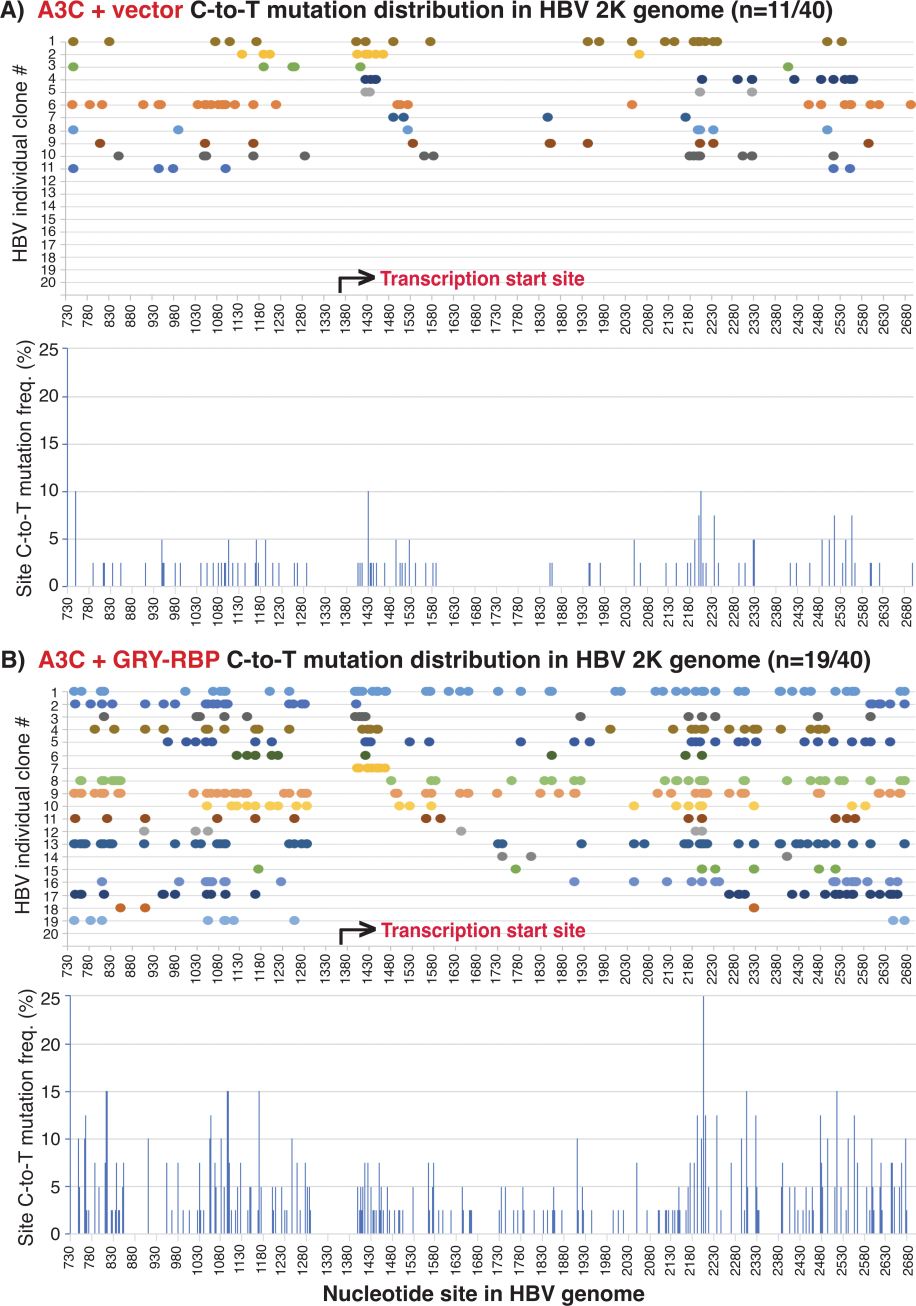
The A1 cofactor GRY-RBP dramatically increases A3C clonal mutational frequency and efficiency on the HBV 2K major genome variant by sequencing analyses. GRY-RBP was co-transfected with A3C and HBV encoding plasmids into HepG2 cells. After a 48 h transfection, HBV rcDNAs were isolated and HBV genome variants were amplified by PCR-95°C. The resultant genomic amplicons were separated by 1% agarose electrophoresis, and the corresponding HBV 2Ks were isolated and TA-cloned into a pCR4 vector for sequencing analyses. Forty clones were randomly selected for each treatment. The clonal C-to-T mutation distributions are linearly presented as color spots along the line against the corresponding cytidine site in the HBV 2K genome in the top panel with site numbering according to the Gene Bank HBV V01460. In addition, the mutation positive clones for each treatment were also combined to evaluate the site mutational frequencies for each cytidine in the HBV 2K genome. The cytidine site mutational frequencies represent the number of C-to-T mutations for a specific cytidine site in the mutation positive clones divided by the sequenced clone number (*n* = 40) and are presented as a percentage bar against each cytidine site in the HBV 2K genome in the lower panel. The side-by-side comparison data are presented for the treatment of (A) A3C + Vector and (B) A3C + GRY RBP. The arrow in each panel indicates the reverse transcription start site of HBV genome during viral replication in the capsids.

HBV 2K is the major HBV genome variant with an internal 2697–3182/1-736 nt deletion during HBV viral replication in HepG2 cells (14). Forty clones were randomly selected from each treatment of vector control, A3C, A3C + GRY RBP, and A3C + KSRP for sequencing analyses. The C-to-T mutation distributions in each mutation-positive HBV 2K genome clone were presented in Fig. 11 and S4 as color spots along the line against each cytidine site as numbered in the HBV V01460 genomic sequence. A linear 737–2696 nt graphic presentation is used for illustration, although the actual HBV 2K clonal sequence beginning and ending are within 1340–1360 nt due to the PCR primer design and the two internal deletion ends of 737 nt and 2696 nt being covalently connected (see Fig. 2 for its position and structure).

As shown in Fig. 11, A3C over-expression had 11 mutation-positive 2K clones with a total of 125 C-to-T mutations. Co-expression of GRY-RBP with A3C resulted in 19 mutation-positive clones with a total of 459 C-to-T mutations. Most of A3C alone clones had C-to-T mutations starting at or near the HBV reverse transcription start site (1450 nt) and continuing along the reverse transcription reaction to the other termination end with only one clone starting later than 1600 nt. GRY-RBP co-expression had 11 clones with C-to-T mutations starting at or near the HBV reverse transcription start site (1450 nt) with increased C-to-T mutation density. In addition, GRY-RBP co-expression had eight clones with C-to-T mutations starting later than 1600 nt and seven of them had C-to-T mutations continuing along the reverse transcription reaction to the other termination end, indicating that GRY-RBP increased A3C accessibility to the HBV single-stranded (−)DNA and stabilized the A3C mutation reaction along with viral reverse transcription. As shown in Fig. 11 lower panels, A3C had collectively similar site mutation frequencies from the HBV reverse transcription starting site to the terminal end. However, GRY-RBP co-expression increased A3C site mutation frequencies with gradual increases toward the viral reverse transcription end. A similar C-to-T mutation pattern was also observed by KSRP co-expression (see Fig. S4). These data indicate that these A1 cofactors not only increase A3C accessibility to HBV (−)DNA but also stabilize the A3C mutation reaction complex allowing continuance to the other end.

There are 451 cytidines available for C-to-T mutation in each HBV 2K clone. A3C mutational activity was further evaluated by clonal mutation efficiency, a percentage determined by the C-to-T mutation number in a clone divided by the 451 cytidines available. As summarized in Table 2, A3C alone had clonal C-to-T mutation efficiencies varying from 0.88% to 6.65% with an average of 2.58%. GRY-RBP co-expression increased the A3C average mutational efficiency about 2-fold from 2.58% to 5.32% with the highest being 13.53% as shown in Fig. 11B clone #1, indicating that GRY-RBP co-expression with A3C resulted in hypermutation. Similarly, KSRP co-expression also increased the A3C average clonal mutation efficiency up to 3.22% with the highest being 8.65%. These data demonstrate that GRY-RBP and KSRP significantly increase A3C mutations on the HBV 2K major genome variant and generate kataegis-like hypermutation.

**TABLE 2:** Stimulating effect of A1 cofactors on A3C mutational activities by HBV genome sequencing analyses

HBV target	Treatment	Total sequenced clone number (n)	Mutation positive clone number (x/n)	C-to-T mutational # detected in each individual mutation positive clone	Total C-to-T# detected	Average C-to-T # per clone	Average C-to-T #/total C^*a*^ available per clone (%)	Clonal highest C-to-T #/total C^*a*^ available per clone (%)
2K whole genome	Vector	40	4	14, 13, 8, 7	42	10.50	2.33	3.10
	A3C + Vector	40	11	30, 22, 16, 14, 10, 9, 7, 6, 6, 4, 4	128	11.64	2.58	6.65
	A3C + GRY RBP	40	19	61, 58, 56, 38, 33, 30, 29, 29, 25, 22, 15, 14, 10, 10, 9, 7, 6, 4	456	24.00	5.32	13.53
	A3C + KSRP	40	15	39, 37, 20, 19, 14, 13, 13, 12, 11, 9, 8, 7, 7, 5, 4	218	14.53	3.22	8.65
3K 1360–3130 nt	A3C + Vector	40	8	52, 31, 15, 12, 10, 10, 10, 9	149	18.63	4.65	12.97
	A3C + GRY RBP	40	17	69, 47, 43, 33, 26, 24, 19, 18, 18, 15, 14, 13, 9, 9, 9, 8, 6	380	22.35	5.57	17.21

^*a*^ Total cytidines available for potential mutation in HBV 2K and partial 3K genome are 451 and 401, respectively. Mutational efficiency definition: a percentage of the number of C-to-T mutations in a single mutation positive clone divided by the total cytidines available in the clone.

**Fig 12:**
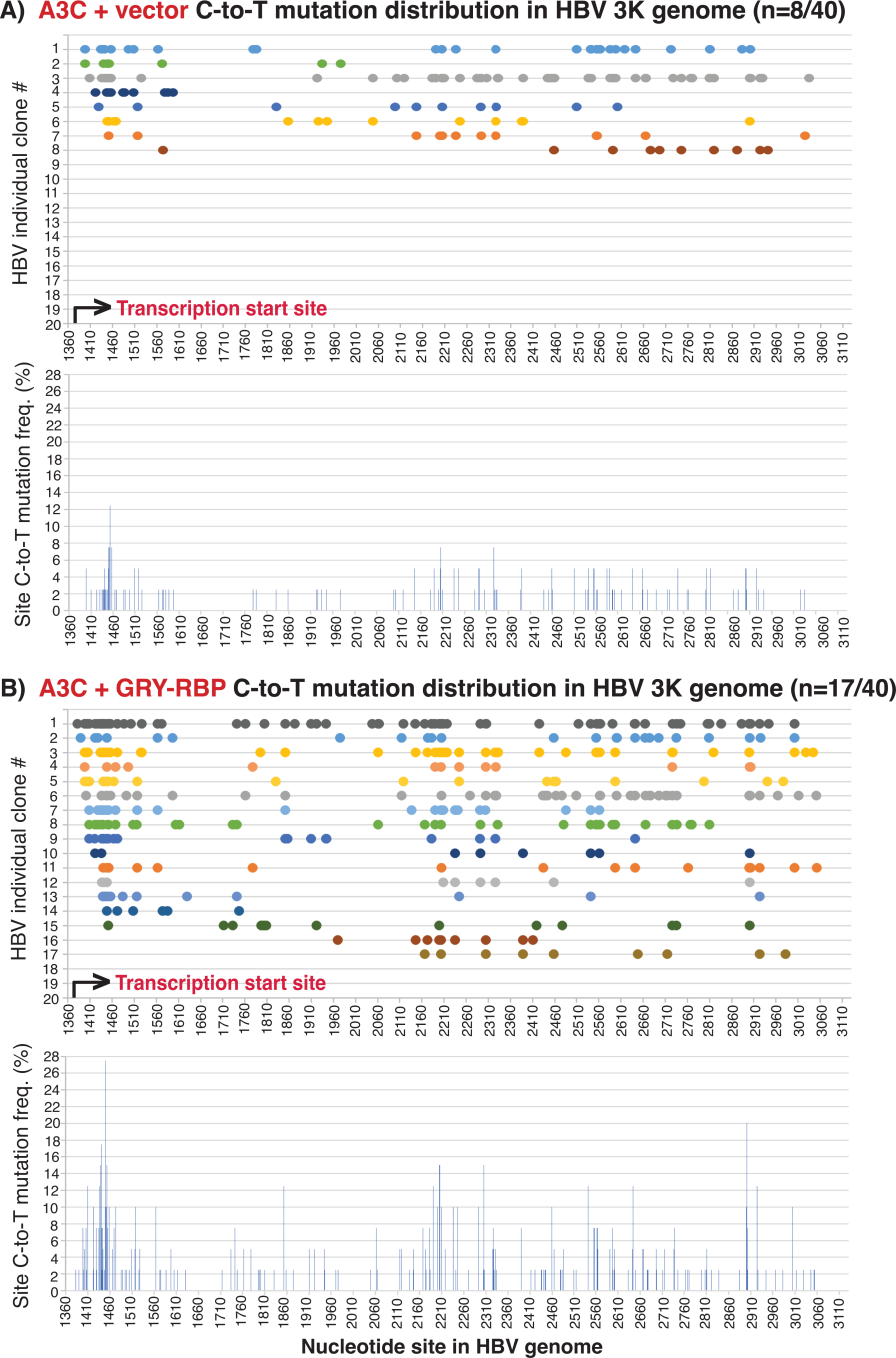
The A1 cofactor GRY-RBP dramatically increases A3C accessibility to HBV 3K full-length genome by sequencing analyses. GRY-RBP was co-transfected with A3C and HBV encoding plasmids into HepG2 cells. After a 48 h transfection, HBV rcDNAs were isolated and the HBV 3K genome region 1360–3130 nt was selectively amplified by PCR-95°C. The resultant amplicons were TA-cloned into a pCR4 vector for sequencing analyses. Forty clones were randomly selected for each treatment. The clonal C-to-T mutation distributions are linearly presented as color spots along the line against the corresponding cytidine site as in the HBV V01460 genome. The mutation positive clones for each treatment were also combined to evaluate the site mutational frequencies for each cytidine in the HBV genome region and are presented as a percentage bar against each cytidine site in the HBV 3K genome region. The data are presented for the treatment of (A) A3C + Vector and (B) A3C + GRY RBP.

A3-induced mutations on HBV full-length 3K genome are unique because the large 3K genome size decreases the available space in the HBV viral capsid. Most of HBV 3K mutations start from the beginning in parallel with the viral reverse transcription initiation site (1450 nt), whereas other HBV genome variants including the major 2K form have C-to-T mutations starting either from the reverse transcription initiation site or downstream (14). To investigate the role of GRY-RBP on A3C-induced HBV full-length 3K mutations, the HBV 3K 1360–3130 nt major beginning region was selectively amplified by PCR-95°C for cloning and sequencing analyses (see Fig. 2 for detailed structure and primer locations). As shown in Fig. 12, GRY-RBP co-expression increased A3C mutation-positive clones from 8 to 17. Importantly, 15 of 17 mutation positive clones by GRY-RBP co-expression had C-to-T mutations starting around the HBV viral reverse transcription initiation site (1450 nt) and then continuing along the chain. As shown in Fig. 12 lower panels, the clonal combined cytidine site mutation frequency showed the highest mutation frequency around 1450 nt but remained lower or had similar levels across the downstream region. These data suggest that GRY-RBP co-expression increases A3C access to the HBV 3K ssDNA from the viral reverse transcription initiation site and generates about 2-fold higher C-to-T mutations in parallel to the viral reverse transcription process. As summarized in Table 2, GRY-RBP co-expression increased the A3C-induced total C-to-T mutation number from 149 to 380 in 40 screened clones with the largest mutational efficiency increase from 13.0% to 17.2%, indicating the presence of hypermutation in HBV 3K genomes. These data provide further evidence that GRY-RBP co-expression increases A3C HBV ssDNA accessibility in the HBV genome and generates hypermutation in parallel to the viral reverse transcription process.

## DISCUSSION

APOBEC-3 (A3) proteins play an important role in host immunity against viruses and human genome mutations in cancer. Although A3s have been investigated extensively, how they are regulated to levels of hypermutation under physiological conditions remain to be elucidated. Unlike APOBEC-1 (A1), A3 proteins alone have deamination activity *in vitro*, suggesting that no cofactors are required for their cellular functions. However, A3s bind quickly to RNAs to form ribonucleoprotein (RNP) complexes once translated in the cellular cytoplasm and multiple hnRNPs have been identified in the A3 complexes (16, 23–26). It is unknown if the A3 complex associated cellular factors could have any role on A3 mutation activity. A3 proteins are single-stranded DNA (ssDNA) specific cytidine deaminases that require access to ssDNA to be functional. For example, it is reported that the Epstein-Barr virus BORF2 directly inhibits A3B to preserve its viral genome integrity through binding to the L1 and L7 regions of A3B C-terminal domain (A3Bctd) that effectively blocks the A3B active site from binding to ssDNA substrates and catalyzing deamination (33). The availability of ssDNA is critical for A3-induced mutations.

Single-stranded DNAs are often only momentarily available during viral reverse transcription or genomic replication and transcription, indicating the presence of cellular factors that help A3s access ssDNA under physiological conditions because A3 induced DNA mutations are not randomly observed. Recently, investigation of A3 induced HBV mutations through HBV whole genome sequencing landscape analyses has shown that A3 mutations predominantly occur on HBV (−)DNA during the process of reverse transcription in viral capsids with significantly varied C-to-T mutation efficiency (14). These data indicate that A3s’ access to HBV ssDNA occurs temporarily during reverse transcription and A3-induced HBV mutations occur along with the reverse transcription reaction. The significantly variable clonal mutational efficiencies also suggest that A3-induced HBV mutational activity is significantly upregulated by the presence of other cellular factors in the viral capsids. Alternatively, these cellular enhancing factors may be insufficient or absent with those clones having lower mutational rates. In this study, we demonstrate that A1 cofactors and other hnRNPs are associated with A3 complexes and upregulate A3 mutational activity to levels of hypermutation, through a mechanism affecting A3s’ ssDNA substrate accessibility and mutation reaction efficiency.

The exact components of the A3 complexes are unknown, although hnRNPs, Hsps, or RNAs were detected in A3 complexes by immunoprecipitations (IP) (16, 23–25). Like those of hnRNPs, we found that A1 cofactors, protein components associating with the A1 apoB mRNA editing complex, were also associated with A3 complexes and that A1 cofactors had stronger protein interactions with A3G than other representative hnRNPs (see Fig. 1). Surprisingly, we found that all known A1 cofactors stimulated A3 mutational activity except RBM47 and that A1 cofactors were more potent than other representative hnRNPs on A3 mutation regulation (see Fig. 3 and 5). This indicates that the regulation by A1 cofactors is a common phenomenon among APOBEC family members. In fact, it has been reported that AID, another member of APOBEC family, was oppositely regulated by hnRNP-Q (alternative name of A1 cofactor GRY-RBP) and hnRNP-L for different functions (34). Like AID, hnRNP-Q increased A3-induced HBV mutational activity, whereas hnRNP-L was also inhibitory as described in this study. Importantly, when their endogenous expression protein levels were knocked-down by siRNA, opposite effects to their over-expression were observed on A3 mutation activity (Fig. 8), indicating that A1 cofactors like hnRNP-Q and representative hnRNPs like hnRNP-L are involved in regulating A3 mutational activity. These data also indicate that there is a complex dynamic interaction amongst multiple components associated with A3, which leads to either stimulating or inhibitory effects *in vivo*.

When the A3 complexes were disrupted through A3G or A3B mutagenesis, A3 mutational activity decreased significantly in association with significantly decreased A3 protein interactions with hnRNPs or HBV Polymerase (Fig. 9 and 10), indicating a requirement of A1 cofactors and hnRNPs for A3 full function. Both A3G stability and mutational activity were compromised when A3G protein interaction with hnRNPs was disrupted by the A3G mutagenesis, indicating a critical role of A1 cofactors and other hnRNPs on A3G function. In addition, A3G lost its HBV mutational activity to almost background levels by the L35A mutant in association with a significantly decreased A3G protein interaction with HBV polymerase, indicating that HBV polymerase may also play an important role for A3G to access HBV (−)DNA during reverse transcription and move along the reverse transcription chain reaction. On the other hand, A3B stability was not affected (Fig. 10) even when the A3B complex was dissociated completely through the A3B 4Y/A + W127A mutation (24). However, A3B mutant induced HBV C-to-T mutational activity was also decreased significantly, which could not be restored by GRY-RBP co-expression (Fig. 10), indicating that A3B protein interactions with hnRNPs were also essential for its function. Together, these data provide additional evidence that A3G and A3B loss their enzyme activities without these cellular factors and A1 cofactors and hnRNPs are essential components involved in A3 function and regulation under physiological conditions.

Co-expression of the A1 cofactor, GRY-RBP increased A3C-induced mutations up to 3.5-fold in the total C-to-T mutation number by HBV major genome 2K variant sequencing analyses (see Table 2). GRY-RBP co-expression not only increased the A3C mutational frequency as shown in the increased mutation-positive clone number but also increased the cytidine mutation efficiency in individual clones (Fig. 11). Importantly, GRY-RBP co-expression increased A3C clonal cytidine mutational efficiency 2-fold in the most effective clonal mutation reaction from 6.65% to 13.53%, indicating that A3C achieved a much higher mutational efficiency by GRY-RBP co-presence and stimulation in HBV viral capsid. In addition, A3G has a strong protein interaction with A1 cofactors including GRY-RBP (see updated Fig. 1C), and over-expression of A1 cofactors or hnRNPs alone had no effect on HBV genome mutation, although they increased HBV nucleocapsid production levels (see Fig. S3). These data suggest that GRY-RBP co-packages with A3C in the HBV viral nucleocapsid to significantly upregulate A3 C-to-T mutational activity on the HBV viral genome and lead to Kataegis-like hypermutation as observed in mutations associated with human cancer.

On the other hand, GRY-RBP co-expression with A3C resulted in a unique C-to-T mutation distribution pattern among HBV full-length 3K genome mutation-positive clones (see Fig. 12). Almost all these clones had C-to-T mutations starting around the HBV viral reverse transcription initiation site (1450 nt) and then continuing towards the other viral reverse transcription end with lower or collectively similar site cytidine mutation frequencies for the downstream chain reaction, indicating that GRY-RBP co-expression increased A3C accessibility to the HBV 3K ssDNA, leading to >2-fold mutation increase from the beginning of the viral reverse transcription. These data demonstrate that GRY-RBP not only increases A3C accessibility to the single stranded (−)DNA from the beginning of HBV reverse transcription but also stabilizes the A3C mutation reaction complex that is moving along with the HBV reverse transcription chain reaction.

It is interesting to compare the effectiveness of A1 cofactors on different A3s to the A3s’ protein interaction strength with hnRNPs. A1 cofactors had different stimulating effects on different A3s with a general order of A3C > A3G>A3B (Fig. 3 and 5), which is opposite to the A3s’ hnRNP protein interaction strength order of A3B > A3G>A3C (26). A3B has strong hnRNP binding ability but was less sensitive to A1 cofactors’ co-expression. On the other hand, hnRNPs were essential for A3B function as revealed by the A3B mutagenesis results in Fig. 10, and GRY-RBP partially restored the lost A3B mutation activity. These data indicate that A3B is less sensitive to additional A1 cofactors or other hnRNPs probably because A3B already exists as RNP complexes, interacting with hnRNPs already expressed in cells. In addition, the pre-existing hnRNP association could be the reason that A3B has a stronger mutational activity than other A3s under conditions like cancer.

Functioning as critical alternative splicing regulators, hnRNPs have been implicated in various aspects of cancer (35, 36). HnRNP-A1 and hnRNP-AB (ABBP1) are highly expressed in most tumors. HnRNPs participate in cancer-related pathways including DNA repair and IL6/JAK/STAT3 signaling. HnRNP-K was identified as a tissue biomarker for detection of early hepatocellular carcinoma (HCC) (37). Consistent with these observations, we also found that the hnRNP A1 cofactors, GRY-RBP (hnRNP-Q) and ABBP1 (hnRNP-AB), stimulated A3 mutational activity to generate hypermutations. It has been reported that when human cancer cell lines were cultured for extended periods to investigate ongoing patterns of mutation generation, signatures of A3 induced genome mutations displayed substantial fluctuation in mutation rates over time with episodic bursts of mutations (15). The initiating factors for these bursts were unknown and not associated with the expression levels of A3s. Our data reported here suggest that the episodic bursts of mutations observed in the cell line cultures could be associated with up-regulation of A3 mutational activity through potential modulation of cellular factors like A1 cofactors or hnRNPs. On the other hand, characterization of full-length genomes of HBV quasispecies in sera of patients at different phases of infection has shown that patients with acute-on-chronic liver failure had higher heterogeneity, and the mutations in the Core promoter and pre-Core region were the highest with a kataegis-like localized hypermutation pattern. Although there were no reports of A3 and hnRNP expression levels with these patients, our study suggests that these acute-on-chronic liver failure patients could have increased expression of A3s, A1 cofactors, or other hnRNPs that led to significantly increased A3 activity to generate HBV hypermutation. As previously suggested (7, 38), hnRNPs could be a potential therapeutic target affecting HBV replication or cancer associated with A3-induced mutations.

In summary, we investigated the possible role of A1 cofactors and other representative hnRNPs on A3 hypermutation, using the HBV cellular replication system in HepG2 cells. We found that A1 cofactors and other hnRNPs are involved in regulating A3 mutational activity. Endogenous gene expression knockdown by siRNA had the opposite effect on A3 mutational activity compared with their gene over-expression. Disruption of the A3 complex association with hnRNPs through A3G and A3B mutagenesis diminished its mutational activity, demonstrating that hnRNPs are essential components for A3 mutational function. A1 cofactors like GRY-RBP can not only increase A3C mutation frequency in the HBV genome population but also increase individual mutation reaction efficiency through increasing A3C (−)DNA accessibility and mutation chain reaction stability to generate kataegis like bursting hypermutation. These findings provide evidence that A1 cofactors and other hnRNPs may play an important regulatory role under physiologic and pathophysiologic conditions.

## MATERIALS AND METHODS

### Plasmid DNA constructs

The plasmid encoding the HBV viral genome was a gift from Dr. Josef Kock (30). It was generated from an HBV *ayw* strain having a sequence identical to HBV V01460 in the gene bank. HBV Polymerase and Core protein genes were PCR amplified from the HBV encoding plasmid by AccuPrime Pfx DNA polymerase (Thermo Fisher Scientific) using primers containing appropriate restriction enzyme sites in the end. Both HBV polymerase and core protein were constructed with a FLAG tag (DYKDDDDK) immediately after the C-terminal amino acid and cloned into pcDNA3.2 expression vector through an intermediate vector pENTR1A (Thermo Fisher Scientific) according to the manufacturer’s instructions.

A3C and A3G were RT-PCR-amplified from the human acute monocytic leukemia cell line THP-1 and were cloned into a pcDNA3.2 vector with a 9-amino acid HA tag at the C terminus as previously reported (22, 25). The A3B long form variant was purchased from Origene. The A3B wild-type-pCMV6-XL5 was generated from the A3B long form by internal deletion using the Quick-Change II XL kit (Agilent). A3G or A3B mutants were generated from A3G-pcDNA3.2 or A3B-pCMV6-XL5 using the Quick-Change II XL kit according to the manufacturer’s instructions.

HnRNP-Q6, A1, A2B1, D, E2, F, L, R, and RBM47 in the pCMV6-Entry vector with a FLAG tag at the C terminus were purchased from Origene. ACF, CUGBP2, GRY-RBP, KSRP, hnRNP-C1, ABBP1, ABBP2, and BAG4 in the pcDNA3.2 vector were prepared as previously reported (21, 22).

### Protein interaction analyses

For *in vitro* protein interaction analyses, A1 or A3C was co-translated with A1 cofactors by an *in vitro* coupled transcription/translation system (Promega) in the presence of ^35^S-methionine according to the manufacturer’s instruction. The protein complexes formed during the *in vitro* translation were immunoprecipitated by an antibody against A1 or A3C using Protein G DynaBeads kit (Thermo Fisher Scientific) according to the manufacturer’s instructions. The precipitated protein complexes were separated by a 12% SDS-PAGE denaturing gel, and the ^35^S-labeled proteins were detected by a PhosphoImager.

For cellular protein interaction analyses in 293 FT cells, A3G or A3B were co-transfected with A1 cofactor or hnRNP encoding plasmid in a ratio of 1:1 into 293 FT cells by X-tremeGENE HP. The 293 FT cell line was purchased from Thermo Fisher Scientific and was maintained in RPMI 1640 containing 10% fetal bovine serum. After a 48 h post-transfection, the cells were lysed in a 300 µL buffer containing 50 mm Tris-HCl, pH 8.0, 1.5 mm MgCl_2_, 0.5% Nonidet P-40, and 1× proteinase inhibitor mixtures. The A3G or A3B cellular complexes were immunoprecipitated (IP) by a FLAG antibody (Sigma) against the FLAG tag in the C-terminal of target genes using Protein G DynaBeads kit (Thermo Fisher Scientific) according to the manufacturer’s instructions. For RNase pre-treatment effect on A3B protein interactions, the cell lysate aliquots were incubated with RNase A in a final concentration of 0.10 mg/mL at room temperature for 30 min before the immune-precipitation procedure. The A3G or A3B levels in the different IPs were determined by electrophoresis in a 10% NuPAGE Bis-Tris gel followed by western blotting analyses using an HA antibody against the HA tag in A3G (Sigma) in Fig. 1 and 9 or antibody against A3B (Abcam) in Fig. 10.

For protein interaction analyses in HepG2 cells, A1 cofactors or hnRNPs, A3G, and HBV were transfected at a ratio of 4:1.5:0.5 into HepG2 cells on collagen-coated plates, and the cells were lysed at 48 h after the transfection. The protein interactions were analyzed in the same way as described above.

### Cell culture and extractions of HBV viral rcDNA

The human liver cell line HepG2 was purchased from ATCC and was maintained in EMEM containing 10% fetal bovine serum. For investigating the effects of A1 cofactors or representative hnRNPs on A3-induced HBV viral mutations, A1 cofactor, A3, and HBV-encoding plasmids were co-expressed in HepG2 cells, and the resultant HBV DNAs in the replication intermediate, rcDNA containing capsids, were isolated and analyzed as previously reported (14). Briefly, HepG2 cells were plated on 35 mm 6-well collagen-coated plates the day before transfection. Plasmids encoding A1 cofactor or hnRNP, A3, and HBV in a ratio of 4:1.5:0.5 were mixed and co-transfected into HepG2 cells by X-tremeGENE HP (Sigma). A total of 2 µg of plasmid DNA and 6 µL of transfection reagents were added to HepG2 cells in each well. Three separate samples were prepared for each evaluation. An empty pcDNA3.1 vector was used as a blank control.

For HBV viral rcDNA extractions, HepG2 cells were harvested 48 h post-transfection by cell lysis in a 300 µL buffer containing 50 mm Tris-HCl, pH 8.0, 1.5 mm MgCl_2_, and 0.5% Nonidet P-40. After pretreatment with 10 units of micrococcal nuclease (New England Biolabs) in the presence of 2 mm CaCl_2_ at 37°C for 1 h, the cell cytoplasm lysate was digested with 20 µL of 20 mg/mL proteinase K at 55°C for 15 min, and the HBV rcDNAs released from the viral capsids were isolated by a Pure Link genomic DNA purification kit (Thermo Fisher Scientific) according to the manufacturer’s instructions. Of note, since A3 mutational activities were determined by HBV DNA C-to-T mutations during HBV viral replication after co-transfection in HepG2 cells, all gene over-expression levels and resultant HBV DNA mutation levels were variable depending on incubation time. For consistency and accurate side-by-side comparisons, the transfection of A1 cofactor or hnRNP, A3, and HBV were kept at same ratio of 4:1.5:0.5, and the resultant HBV viral capsids were harvested at 48 h post-transfection. The data were presented in the figures by a representative single experiment with three independent samples for each treatment, although each set of experiments was repeated and confirmed 3–5 times.

#### HBV DNA mutation frequency analyses by primer extension

HBV mutational frequencies were quantitatively determined by the primer extension method as described previously (25, 29). Briefly, HBV DNAs were PCR amplified from HBV rcDNA extracts after various treatments. HBV whole genome variants or selected regional PCRs were performed by Gold AmpliTaq with initial denaturing at 95°C for 15 min, and then, 38 cycles of 95°C × 1 min, 58°C × 1 min, and 72°C × 4 or 2 min, respectively, followed by another hold at 72°C × 7 min. Alternatively, for some of applications, HBV DNA regional PCRs were also performed by regular AmpliTaq with initial denaturing at 94°C for 2 min, and then, 38 cycles of 94°C × 30 sec, 58°C × 1 min, and 72°C × 2 min followed by another hold at 72°C × 7 min. The 94°C PCRs were diluted 1:10, and a 2 μL aliquot was taken for further mutated HBV DNA enrichment by 3D-PCR using the same primers as for PCR-94°C on a RoboCycler (Stratagene). The 3D-PCR was performed by initial denaturing at 88°C for 5 min, followed by 35 cycles of 1 min at the denaturing temperature, 1 min at 58°C, 2 min at 72°C, and a final 7 min extension at 72°C.

For HBV mutational frequency analyses by primer extension (pe), PCR amplicons were purified by a QIAEX II gel extraction kit (Qiagen) to remove the dNTP from the PCR reaction. An aliquot of the purified DNA amplicons was subjected to a thermal cycle primer extension reaction in the presence of ddGTP with a 5′-^32^P-end-labeled primer against the selected cytidine at sites 1453, 1664, 1674, or 2564, which were named as pe1453, pe1664, pe1674, or pe2564, respectively (see Fig. 2 for primer locations and sequences in detail). The primer extension reactions were performed by 5 min at 95°C followed by 50 cycles of 45 s at 95°C, 30 s at 56°C, and 45 s at 72°C, and a final 4 min at 72°C. The ^32^P-labeled primer extension products were separated by electrophoresis in an 8% polyacrylamide-urea sequencing gel and quantitated by a PhosphorImager. This method represents a single Sanger sequencing reaction of DNA template against cytidine using the regular DNA sequencing kit. Primer extension stopped at the target cytosine site due to ddGTP incorporation. When the target cytosine was mutated, the primer extension would continue through the site until the next cytosine downstream and resulted in longer oligonucleotide products on the sequencing gel. The HBV mutational frequency at the target cytosine site was determined by the percentage of these bands above the target cytosine site divided by the total product counts in the lane. The final quantification of this method is based on the reading of ^32^P radioactivity of primer-extended products by Phospho-Imager, which provides a good linear range for a side-by-side comparison of multiple samples.

### siRNA-mediated mRNA knockdown analyses

For siRNA knockdown of endogenous A1 cofactors or other hnRNPs, Dharmacon siGENOME SMARTpool siRNA (20 nM) against A3C, CUGBP2, GRY-RBP, ABBP1, hnRNP-A1, and hnRNP-L were transfected into HepG2 cells by Lipofectamine RNAiMax, using a reverse transfection method according to the manufacturer’s instruction. After a 24 h siRNA transfection, the siRNA-containing medium was replaced by fresh medium, and the HepG2 cells were additionally transfected with the HBV viral genome-encoding plasmid with or without A3G or A3C. After 48 h of HBV transfection, the cells were lysed by the 0.5% NP-40 buffer for HBV rcDNA isolation as described above. For protein expression analyses, the cells were lysed by the 0.5% NP-40 buffer containing proteinase inhibitor mixtures (Roche) or by the SDS sample buffer. The expressed protein levels were determined by electrophoresis in a 10% NuPAGE BisTris gel followed by western blotting analyses.

For mRNA expression analyses, the cells were lysed by Trizol reagent (Thermo Fisher Scientific) and total RNAs were isolated according to the manufacturer’s instructions. After being treated by DNase-I treatment at 37°C for 1 h, aliquots of the total RNAs were reverse-transcribed into cDNA by random primer. The expression levels of all genes were determined by quantitative RT-PCR using StepOne Plus (Thermo Fisher Scientific) according to the manufacturer’s instructions. Primers used for qPCR analyses are listed in Table S1.

### HBV DNA mutation analyses by cloning and sequencing

HBV regional or whole genome DNAs were amplified by PCR-95°C for cloning and sequencing analyses with a prolonged 95°C denaturation as previously reported (14). Briefly, HBV whole viral genomes were PCR amplified by Gold AmpliTaq using primers against a select region or the flank region of the HBV replication gap (see Fig. 2 for HBV genome structure and primer locations in detail). The PCRs were performed by initially denaturing at 95°C for 15 min and then 38 cycles of 95 °C × 1 min, 55 °C × 1 min, and 72 °C × 4 min followed by another hold at 72 °C × 7 min. The resultant PCR amplifications were separated by 1% agarose gel electrophoresis, and the bands corresponding to the HBV regional PCR or the 2K HBV genome were cut out and purified by a QIAEX II gel extraction kit (Qiagen). 3’-dA was then added to the gel purified PCR amplicons by incubating with 1U regular AmpliTaq at 72°C for 20 min and cloned into a pCR4 vector by using a TOPO TA-cloning kit (Thermo Fisher Scientific) following the manufacturer’s instructions. A total of 40–60 clones were randomly selected and sequenced by Sanger sequencing. C-to-T mutations in each HBV DNA clone were identified by comparison with the HBV genome V01460 using the online program BLAST. The sequencing information was presented as a C-to-T mutation distribution against the cytidine sites in the HBV V01460. For evaluation of A3 mutational activity, the mutation efficiency for an individual HBV genome clone was calculated by a percentage of the total C-to-T mutation number in the clone divided by the total cytidine number (451 for HBV 2K) available for a potential mutation reaction (Tables 1 and 2).

## Data Availability

The summative data of HBV clonal sequencing analyses by Sanger sequencing methods are presented in Fig. 4, 7, 11 and 12 and Tables 1 and 2. The original raw sequence data can be provided if requested by contacting Zhigang Chen, Clinical Center, National Institutes of Health (chenz2@cc.nih.gov). The rest of the data is presented within the article.

## SUPPLEMENTAL MATERIAL

Supplemental material jvi.01879-24-s0001.pdf Figures S1 to S4 and Table S1.

ASM does not own the copyrights to Supplemental Material that may be linked to, or accessed through, an article. The authors have granted ASM a non-exclusive, world-wide license to publish the Supplemental Material files. Please contact the corresponding author directly for reuse.
