# Supplementary material for: APOBEC-1 cofactors regulate APOBEC3-induced mutations in hepatitis B virus
Source: J Virol. 2025 Jan 27;99(2):e01879-24. doi: 10.1128/jvi.01879-24 (PMC11853063; doi:10.1128/jvi.01879-24)
Supplement: Supplemental material — Figures S1 to S4 and Table S1. [file jvi.01879-24-s0001.pdf]

Supplementary Material to
"APOBEC-1 cofactors regulate APOBEC3 induced-mutations in hepatitis B virus"
by Zhigang Chen et al.

Contents:

Supplemental Figure legends

Figure S1. Gene over-expression profile for co-expression of A1 cofactors with A3C, A3G, or A3B by quantitative RT-PCR analyses

Figure S2. Gene over-expression profile for co-expression of representative hnRNPs with A3C, A3G, or A3B by quantitative RT-PCR analyses

Figure S3. Effect of A1 cofactors or hnRNPs alone vs their co-expression with A3G on HBV viral replication and DNA mutation analyses

Figure S4. A1 cofactor KSRP increases A3C clonal mutational frequency and efficiency on the HBV 2K major genome variant as determined by sequencing analyses

Table S1. Primers used for quantitative RT-PCR

Supplemental Figure Legends

Fig. S1. Gene over-expression profile for co-expression of A1 cofactors with A3C, A3G, or A3B by quantitative RT-PCR analyses

A1 cofactors were co-transfected with A3 and HBV encoding plasmids into HepG2 cells under the same plasmid ratio and condition as in Figs. 3 and 5. After 48 h post transfection, cells were lysed by Trizol reagent and total RNAs were isolated. After being pre-treated with DNase, aliquots of the total RNAs were reverse transcribed into cDNAs by random primers. All gene over-expression levels were determined by quantitative PCR. **A)** A1 cofactor and A3 mRNA over-expression levels relative to their endogenous gene expression in HepG2 cells. **B)** A1 cofactor and A3 mRNA over-expression levels normalized to the endogenous housekeeping gene, GAPDH expression in HepG2 cells. **C)** Effect of A1 cofactor and A3 co-expression on HBV viral RNA production in HepG2 cells. Graph bar values are means $\pm$ SE of 3 independent samples. Blank, background control by mock vector without A3 co-transfection. Vector, A3 alone control with mock vector to balance the total plasmid DNA amount for cell transfection.

Fig. S2. Gene over-expression profile for co-expression of representative hnRNPs with A3C, A3G, or A3B by quantitative RT-PCR analyses

Representative hnRNPs were co-transfected with A3 and HBV encoding plasmids into HepG2 cells under the same plasmid ratio and condition as in Figs. 3 and 5. After 48 h post transfection, cells were lysed by Trizol reagent and total RNAs were isolated. After being pre-treated with DNase, aliquots of the total RNAs were reverse transcribed into cDNAs by random primers. All gene over-expression levels were determined by quantitative PCR. **A)** hnRNP and A3 mRNA over-expression levels relative to their endogenous gene expression in HepG2 cells. **B)** hnRNP and A3 mRNA over-expression levels normalized to the endogenous housekeeping gene, GAPDH expression in HepG2 cells. **C)** Effect of hnRNP and A3 co-expression on HBV viral RNA production in HepG2 cells. Graph bar values are means $\pm$ SE of 3 independent samples. Blank, background control by mock vector without A3 co-transfection. Vector, A3 alone control with mock vector to balance the total plasmid DNA amount for cell transfection.

Fig. S3. Effect of A1 cofactors or hnRNPs alone vs their co-expression with A3G on HBV viral replication and DNA mutation analyses

Representative A1 cofactors and hnRNPs were co-transfected with A3G and HBV encoding plasmids into HepG2 cells under the same plasmid ratio and condition as in Figs. 3 and 5. After 48 h post transfection, cells were lysed for total RNA or HBV rcDNA isolations. **A)** Effect of representative A1 cofactors and hnRNPs alone or their co-expression with A3G on HBV viral replication. After being pre-treated with DNase, aliquots of the total RNAs were reverse transcribed into cDNAs by random primers and HBV viral RNA expression relative levels were determined by quantitative PCR. Alternatively, the production levels of HBV rcDNA in viral capsids were also determined by quantitative PCR using the same primers as HBV viral RNA quantitation. The data are presented as graph bars $\pm$ SE of 3 independent samples for HBV RNA in the left and HBV rcDNA in the right panel. **B)** Effect of representative A1 cofactors and hnRNP alone or their co-expression with A3G on HBV DNA mutation. HBV genome variants' common region 1360-2620 nt were amplified by PCR-95°C. After removal of dNTP, HBV C-to-T mutation frequencies at cytidine sites 1453 in the PCR amplicons were determined by a primer extension analysis, called pe1453. The ^{32}P-labelled primer extension products were separated by

an 8% polyacrylamide sequencing denaturing gel followed by PhosphoImager quantitation for mutation frequencies. The data are presented with the primer extension product gel analyses in the left panel and a mutation frequency graph in the right panel. Graph bar values are means ± SE of 3 independent samples. Vector, background control was determined by mock vector without A3 co-transfection.

Fig. S4. A1 cofactor KSRP increases A3C clonal mutational frequency and efficiency on the HBV 2K major genome variant as determined by sequencing analyses
KSRP was co-transfected with A3C and HBV encoding plasmids into HepG2 cells as in Fig. 11. After a 48h transfection, HBV rcDNAs were isolated and HBV genome variants were amplified by PCR-95°C. The resultant genomic amplicons were separated by 1% agarose electrophoresis and the corresponding HBV 2Ks were isolated and TA-cloned into a pCR4 vector for sequencing analyses. Forty clones were randomly selected for each treatment. The clonal C-to-T mutation distributions are linearly presented as color spots along the line against the corresponding cytidine sites in the HBV 2K genome in the top panel with site numbering according to the Gene Bank HBV V01460. In addition, the mutation positive clones for each treatment were also combined to evaluate the site mutational frequencies for each cytidine in the HBV 2K genome. The cytidine site mutational frequencies represent the number of C-to-T mutations for a specific cytidine site in the mutation positive clones divided by the sequenced clone number (n=40) and are presented as a percentage bar against each cytidine site in the HBV 2K genome in the lower panel. The side-by-side comparison data are presented for the treatment of **A)** Vector, background control and **B)** A3C + GRY-RBP co-expression. The arrow in each panel indicates the reverse transcription start site of HBV genome during viral replication in the capsids.

A) A3/A1 Cofactor co-expression mRNA levels relative to endogeneous expression in fold

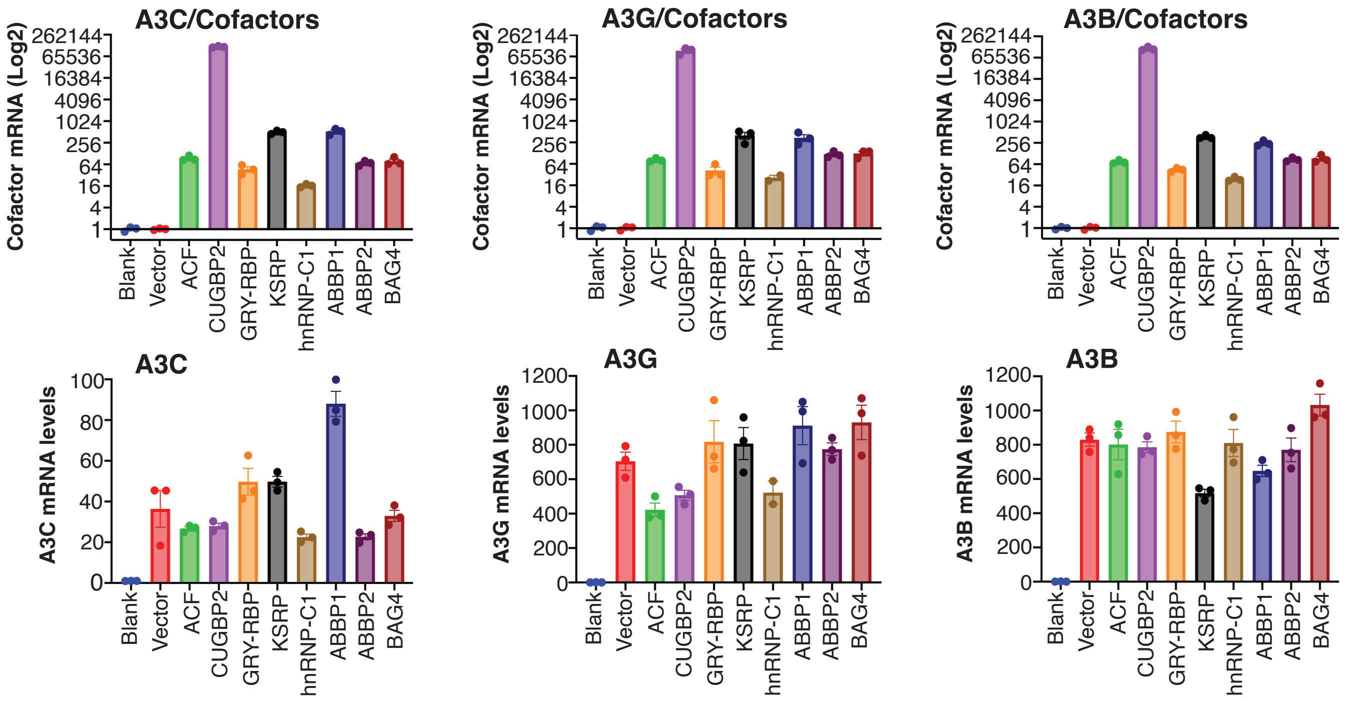

B) A3/A1 Cofactor co-expression mRNA levels normalized to GAPDH expression in fold

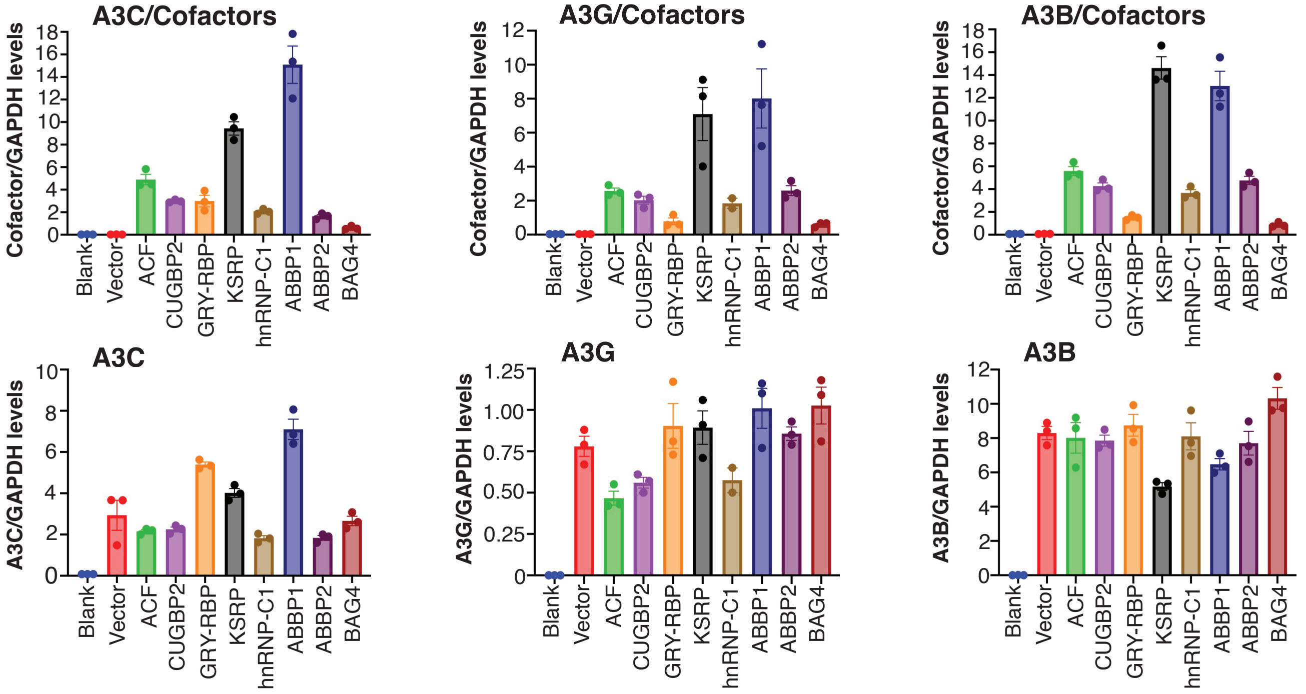

C) A3/A1 Cofactor co-expression effect on HBV RNA expression levels in fold

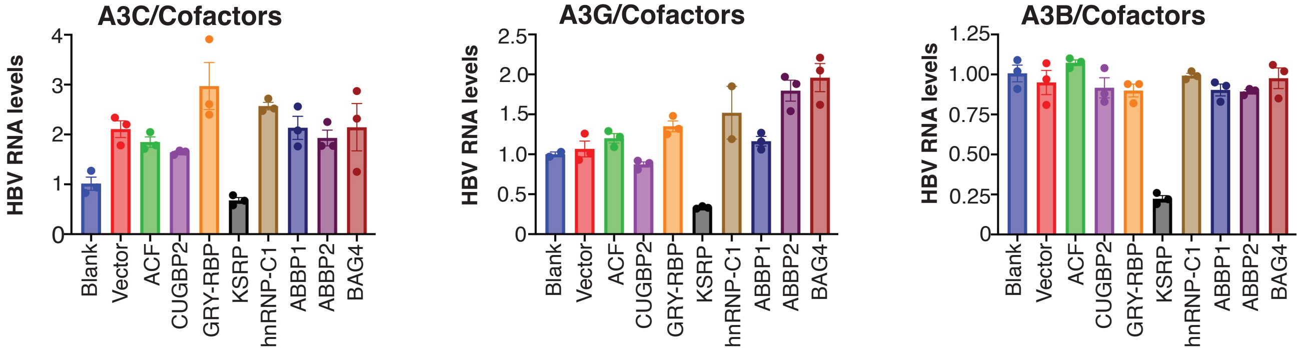

Z. Chen et al., Figure S1

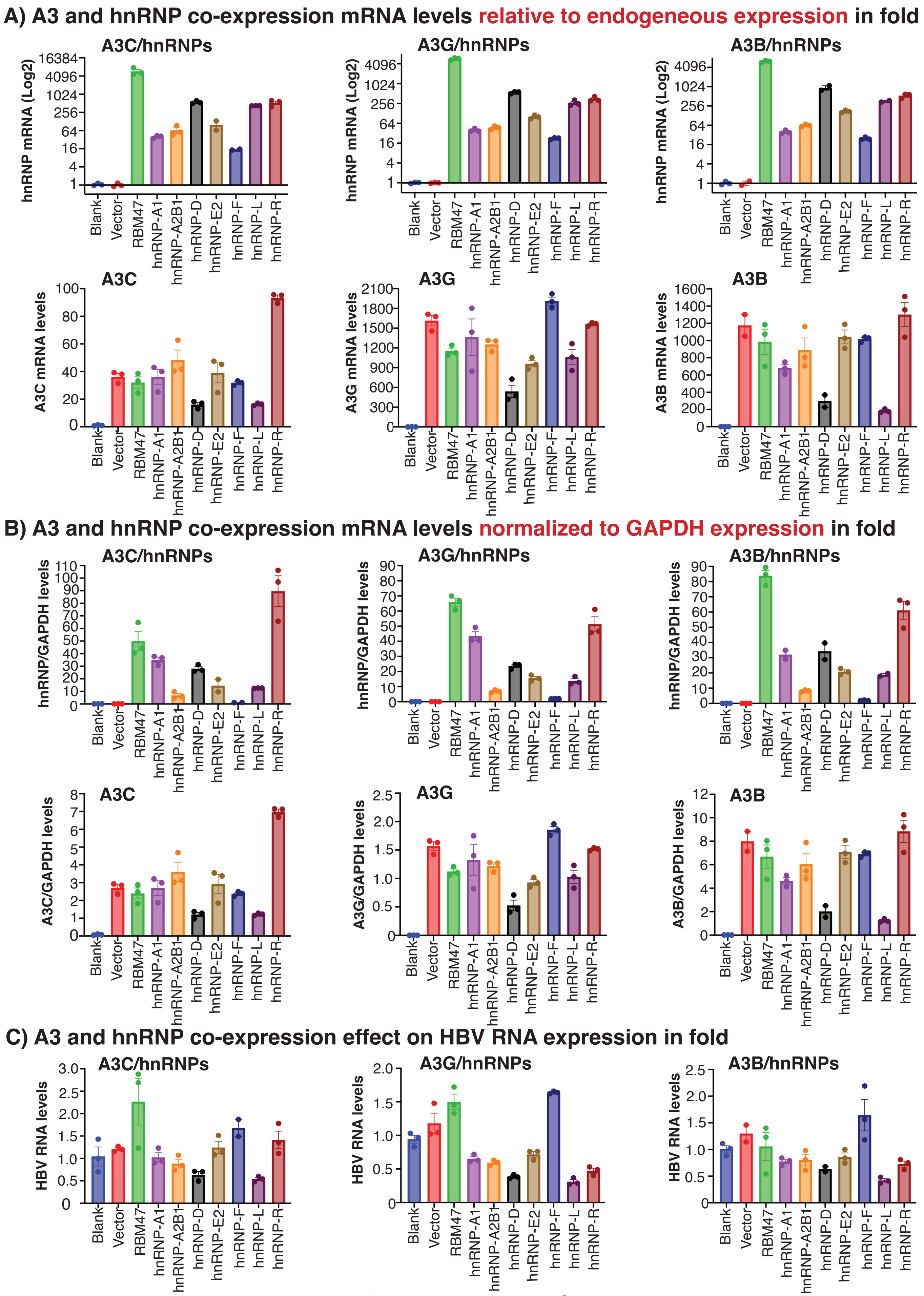

Z. Chen et al., Figure S2

A) Effect of representative A1 cofactors and hnRNPs ± A3G on HBV viral replication

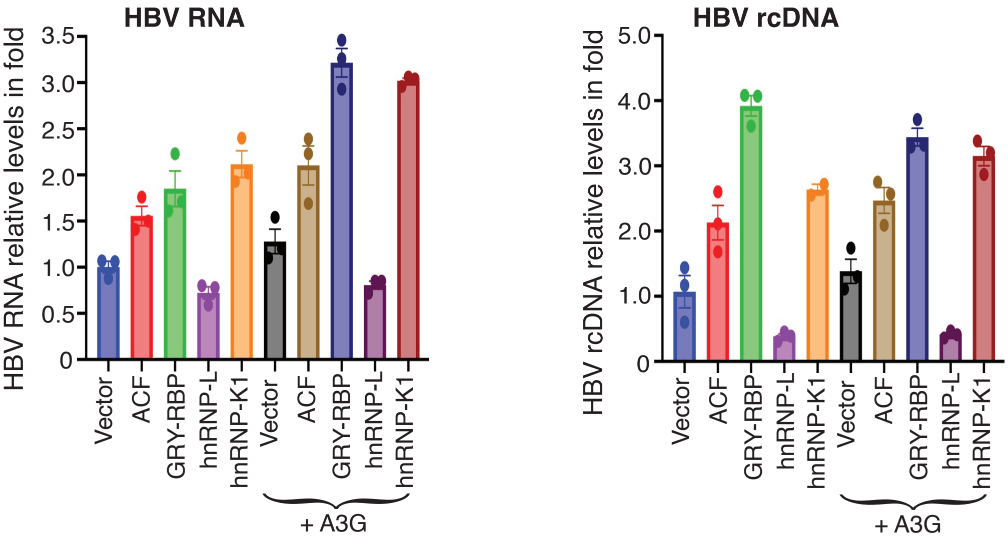

B) Effect of representative A1 cofactors and hnRNP ± A3G on HBV mutational activity by PCR-95°C pe1453

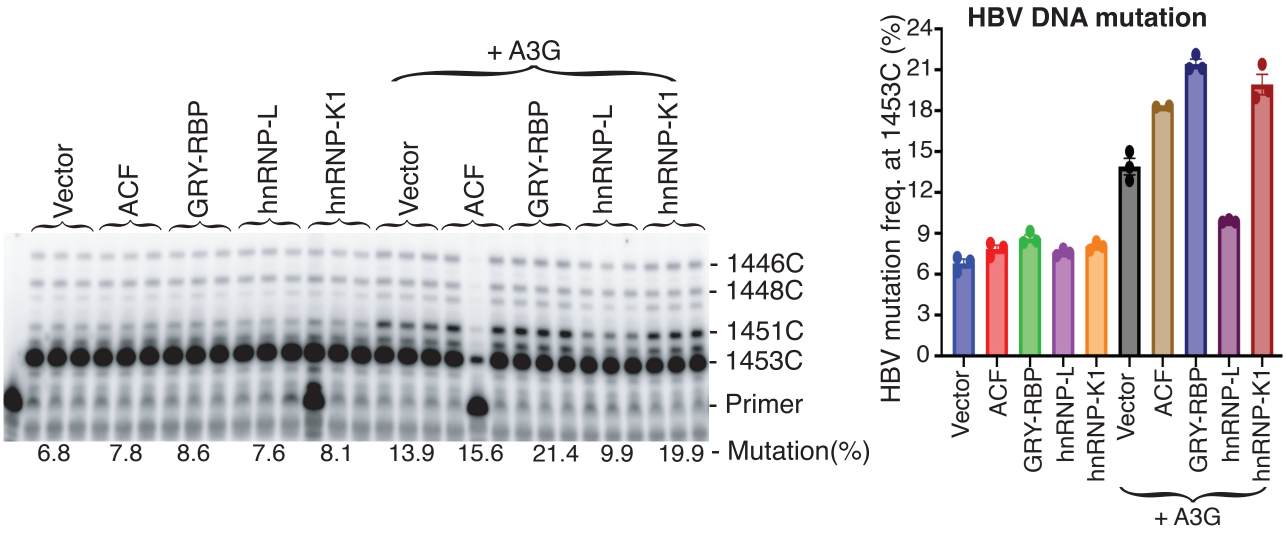

Z. Chen et al., Figure S3

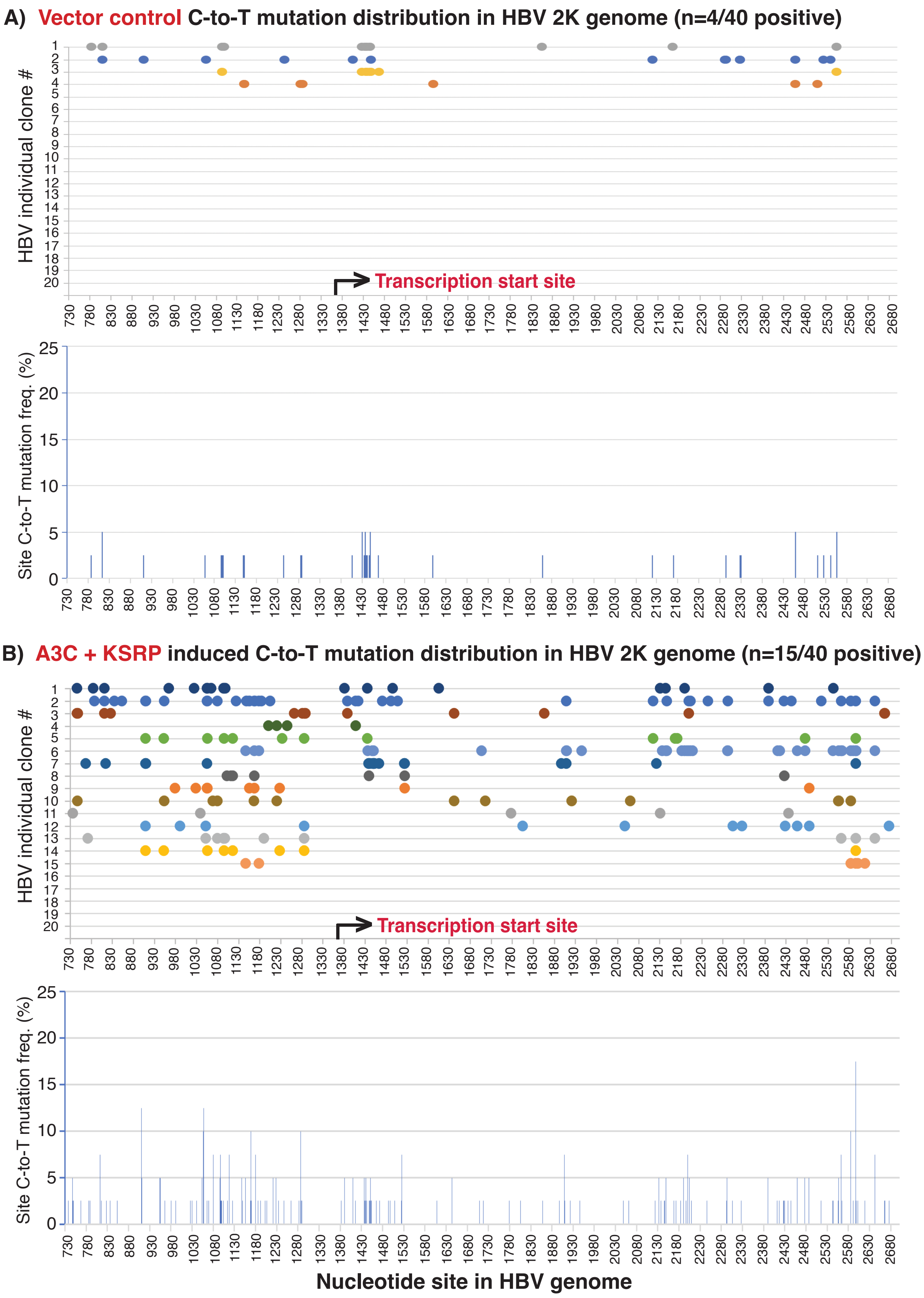

Z. Chen et al., Figure S4

Table S1. Quantitative PCR primers and sequences

Primer name	Primer sequence	PCR size (bp)
ACF-F1	AGAAATGGGCGCCTCTTAGG	
ACF-R1	TATCTGCAGCGCTTGGGTAG	160
CUGBP2-F2	AGATGCAGCAGCTCAACACT	
CUGBP2-R2	TCCATTCAGAGCCGCCATAC	431
hnRNP-Q1-F1	TGGTGCTGTCAAGGCTATGG	
hnRNP-Q1-R1	GTTGGAGGGGGCATATGAGG	183
KSRP-F1	CCTGCTCCTCATGACCCAAG	
KSRP-R1	AAGCCTTCGTGTAGTCCTGC	274
hnRNP-C1-F1	GCAGAGCCAAAAGTGAACCG	
hnRNP-C1-R1	ACGTTTCGAGGGCACTACAG	219
ABBP1-F1	TGGACGGTCAAGAGGGTTTG	
ABBP1-R1	TCTCCCCAAACTCGCCAAAG	218
ABBPP2-F1	CCGGAACCCTGATGATCCAC	
ABBPP2-R1	GACGAGGGGTTCCTCCAAAC	203
BAG4-F1	TATCCTCTTCGCCCTGAACC	
BAG4-R1	AGGAGCCCTAGATCTCGCAG	228
RBM47-F1	AGATTGCCAAGGTCACCGAG	
RBM47-R1	CTCGTCCACGTCGATCTCAG	209
hnRNP-A1-F1	TCCTAAAGAGCCCGAACAGC	
hnRNP-A1-R1	GGCCTTGCATTCATAGCTGC	210
hnRNP-A2B1-F1	TGGACGTGGATTTGGGGATG	
hnRNP-A2B1-R1	CCCATGTTCCTGCTACCACC	276
hnRNP-D-F1	GACCAATAAGAGGCGTGGGT	
hnRNP-D-R1	CTGCTGGTCACCACCTCTTC	208
hnRNP-E2-F1	AGCTCTCCGGTCATCTTTGC	
hnRNP-E2-R1	TCCAAACCTGCCCAATAGCC	287
hnRNP-F-F2	AAGAGTGACGGGTGAAGCAG	
hnRNP-F-R2	TGCTGTTCTGCCCACTGTAG	257
hnRNP-L-F2	CTCAGTGGACAAGGTGACCC	
hnRNP-L-R2	CACTGGTGGACCCATCCTTC	180
hnRNP-R-F1	TGTGGAAAGGAAGCTGCACA	
hnRNP-R-R1	AGGGTCAGCCCATTCAACTG	345
A3C-F1	TCTCTTGGTTCTGCGACGAC	
A3C-R1	AGTCCATGATCTCCACAGCG	234
A3G-F1	AGCCTCACTTCAGAAACACAG	
A3G-R1	TCCTCCACTTGCTGAACCAG	231

A3B-F1	TGACCCTTTGGTCCTTCGAC	
A3B-R1	CTGCAAAGAAGGAACCAGGTC	187
GAPDH-F1	ACCACAGTCCATGCCATCAC	
GAPDH-R1	GTCCACCACCCTGTTGCTGTA	453
